# Quantitation of PEMT and Kennedy Pathways in Liver Phosphatidylcholine Biosynthesis Using NMR

**DOI:** 10.1021/acs.analchem.6c02190

**Published:** 2026-08-29

**Authors:** Ji Yun Lee, Tin Tin Manh Nguyen, Thi Ha Nguyen, Eunbyul Cho, Seungjoon Lee, Hyuk Nam Kwon, Lak Shin Jeong, Dnyandev B. Jarhad, Jin Wook Cha, Sunghyouk Park

**Affiliations:** † Natural Products Research Institute, College of Pharmacy, 26725 Seoul National University, Seoul 08826, Republic of Korea; ‡ Department of Biological Sciences, 35029 University of Ulsan, Ulsan 44610, Republic of Korea; § Research Institute of Pharmaceutical Sciences, College of Pharmacy, 26725 Seoul National University, Seoul 08826, Republic of Korea; ∥ Future Medicine Co., Ltd, 54 Changup-ro, Sujeong-gu, Seongnam, Gyeonggi-do 13449, Republic of Korea; ⊥ KIST Gangneung Institute of Natural Products, Natural Product Drug Development Division, Center for Natural Product Systems Biology, Gangneung 25451, Republic of Korea

## Abstract

Phosphatidylcholine (PtdCho) is a major phospholipid that regulates diverse cellular processes, including lipid metabolism, signal transduction, and cell proliferation. PtdCho is synthesized through two distinct pathways: the Kennedy pathway and the phosphatidylethanolamine *N*-methyltransferase (PEMT) pathway. Because a quantitative comparison of these pathways is critical for understanding lipid homeostasis, several analytical approaches have been developed to assess their relative activities in biological samples. However, most are limited by their reliance on radiotracers and their low sensitivity. Here, we developed a modified high-sensitivity heteronuclear multiple-bond correlation (HMBC) NMR pulse sequence that distinguishes PtdCho species synthesized via the two pathways within a single experiment using U–^13^C-choline and U–^13^C-ethanolamine. By optimizing band-selective polarization transfer and implementing relaxation-based correction, we accurately quantified the ratio of ^12^C- and ^13^C-labeled *N*-methyl carbons, enabling a precise estimation of pathway contributions. Application of this method to murine primary hepatocytes, nontransformed human hepatocyte cell lines (HL-7702 and NKNT-3), and the human hepatoma cell line (Hep3B) revealed species-specific differences: murine hepatocytes exhibited nearly equivalent activities of the PEMT and Kennedy pathways, whereas human liver-derived cells relied predominantly on the Kennedy route. PEMT overexpression in Hep3B cells significantly boosted the PEMT contribution without affecting proliferation, which might suggest species-specific metabolic regulation rather than a conserved tumor-suppressive role. This HMBC-based strategy allows simultaneous and quantitative assessment of PtdCho biosynthetic fluxes without radiotracers, providing a robust platform for elucidating lipid metabolic reprogramming in liver physiology and cancer.

## Graphical abstract

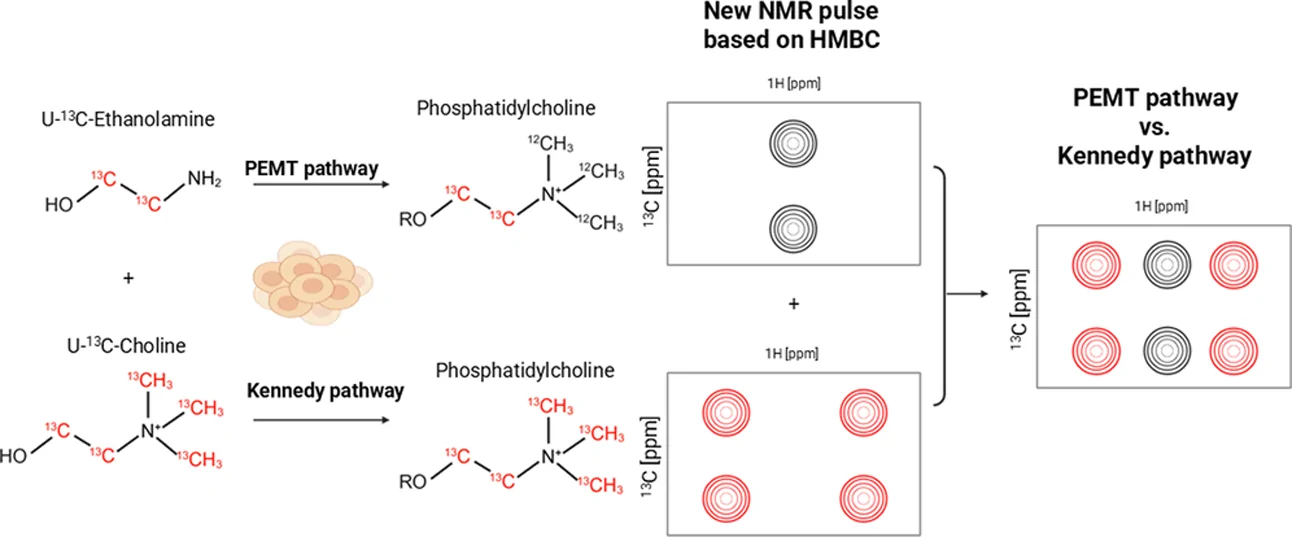

## Introduction

Phosphatidylcholine (PtdCho) is a major phospholipid essential for maintaining cellular membrane integrity in mammalian cells.^1^ It is critical in various cellular processes, including lipid metabolism, signal transduction, and cell proliferation.^1–3^ PtdCho is either obtained through diet or synthesized in the body. For the synthesis in mammalian cells, there are two major de novo pathways: the Kennedy pathway and the phosphatidylethanolamine *N*-methyltransferase (PEMT) pathway. The Kennedy pathway, which synthesizes PtdCho from choline, is usually the larger contributor in most mammalian tissues and relies on dietary choline (Cho) intake. In comparison, the PEMT pathway is active in specific organs, such as the liver (the most active), brain, and adipose tissue, and has been reported to synthesize up to 30% of total PtdCho in the rat liver.^4,5^ The PEMT pathway produces PtdCho by methylating phosphatidylethanolamine (PtdEtha) with methyl groups donated by *S*-adenosylmethionine (SAM), enabling PtdCho synthesis from ethanolamine (Etha) rather than dietary Cho.^1^ It was suggested that the PEMT pathway produces a wider variety of PtdCho species, including longer-chain polyunsaturated forms, while the Kennedy pathway tends to produce more saturated, medium-chain PtdChos.^5^ The activities of the PEMT and Kennedy pathways can also vary significantly among different tissues and developmental stages within the same tissue (e.g., the liver).^6^ Therefore, the two pathways are not completely interchangeable, and they may have specific functional implications in different pathophysiological states. For example, PEMT dysregulation has been linked to diseases such as neurodegeneration, nonalcoholic fatty liver disease (NAFLD), and cancer.^7–10^ Importantly, PEMT has been largely considered a tumor suppressor in the liver based on multiple studies on rat liver.^10–12^ Still, there is a lack of evidence for the causal role of PEMT in human liver cancer tumorigenesis. Therefore, understanding the distinct roles and activities of the Kennedy and PEMT pathways is crucial for elucidating their contributions to various diseases, including liver cancer.

Due to the importance of quantitative comparison of the two pathways, several methods have been developed to measure the activities in biological samples. A traditional approach is to use radiolabeled substrates. For example, methyl-^3^H-SAM, methyl-^14^C-SAM, or ^3^H-Etha is used to assess PEMT activity, whereas methyl-^3^H-Cho is used to assess the Kennedy pathway activity. In previous studies, incorporation of each radiotracer into PtdCho was quantified in independent experiments to assess individual pathway activity.^13–15^ However, the use of radiolabeled substrate is associated with safety and environmental concerns. Thus, recent studies tend to employ mass spectrometry (MS) or ^13^C nuclear magnetic resonance (NMR) combined with nonradioisotopic tracers such as ^2^H or ^13^C-labeled Cho, Etha, and SAM.^4,5,16,17^ Still, MS-based approaches, depending on individual PtdCho species, may have inconsistencies among different PC species, making it difficult to evaluate the overall PC synthesis activity by each pathway. Since individual PtdCho species are differently regulated, PtdCho 34:2 was found to be lower in liver cancer than in adjacent benign hepatic tissues, even though the total PtdCho level, which integrates 46 detected PtdCho species, was not significantly different. Therefore, examining individual PtdCho species might yield different results regarding PtdCho synthesis activity.^18^ In addition, the reported MS-based approach depends on the generation of methyl-D_9_-SAM through the betaine-homocysteine *S*-methyltransferase (BHMT) pathway,^4,5^ which is known to be inactivated in liver cancer,^19–21^ rendering the approach rather inapplicable to liver cancer studies. On the other hand, previous NMR approaches are based on ^13^C detection, which may suffer from sensitivity limitations. Therefore, developing a convenient approach for quantitative dissection between PEMT and Kennedy pathways with higher sensitivity and applicability in liver cancer cells has been desired.

In this study, we propose a new NMR pulse sequence based on HMBC that can distinguish PtdCho synthesized from the two pathways within a single acquisition. We applied it to mouse primary hepatocytes as well as human liver cancer cells and normal liver cells to obtain insights into the differential contributions of PEMT in these cells and the tumor suppressor roles of PEMT.

## Experimental Section

### Synthesis of U–^13^C-Choline Iodide

U–^13^C-choline iodide **2** was synthesized as shown in Scheme 1. No unexpected or unusually high safety hazards were encountered. To a stirred solution of ^13^C_2_-ethanolamine **1** (0.10 g, 1.00 mmol) in anhydrous methanol (5 mL) were added potassium carbonate (0.53 g, 3.85 mmol) and ^13^C-methyl iodide (0.51 g, 3.57 mmol) at room temperature under a nitrogen atmosphere. The resulting reaction mixture was stirred at the same temperature for 12 h. After which, insoluble inorganic salt was filtered on a Celite pad and washed with methanol (5 mL × 2). The filtrate was concentrated under vacuum, and the residue was lyophilized in acetonitrile and water (1:3) to give U–^13^C-choline iodide **2** (0.23 g, 97%) as a pale yellow solid. U–^13^C-choline iodide **2** was characterized by NMR spectroscopy using a JEOL JNM-ECZ400S 400 MHz NMR spectrometer (JEOL Ltd., Tokyo, Japan). The corresponding spectra and assignment table are provided in the Supporting Information. ^1^H NMR (400 MHz, CD_3_OD): δ 4.17–4.14 and 3.82–3.78 (m, together 2H), 3.75–3.65 and 3.32–3.30 (m, together 2H), 3.41–3.38 and 3.08–3.00 (t, together 9H, *J*_13C_–_1H_ = 148 Hz, *J*_2_ = 3.2 Hz). HRMS (FAB+) *m*/*z*: [M]^+^; Calcd for ^13^C_5_H_14_NO^+^ 109.1242; Found 109.1251.

**Scheme 1:**
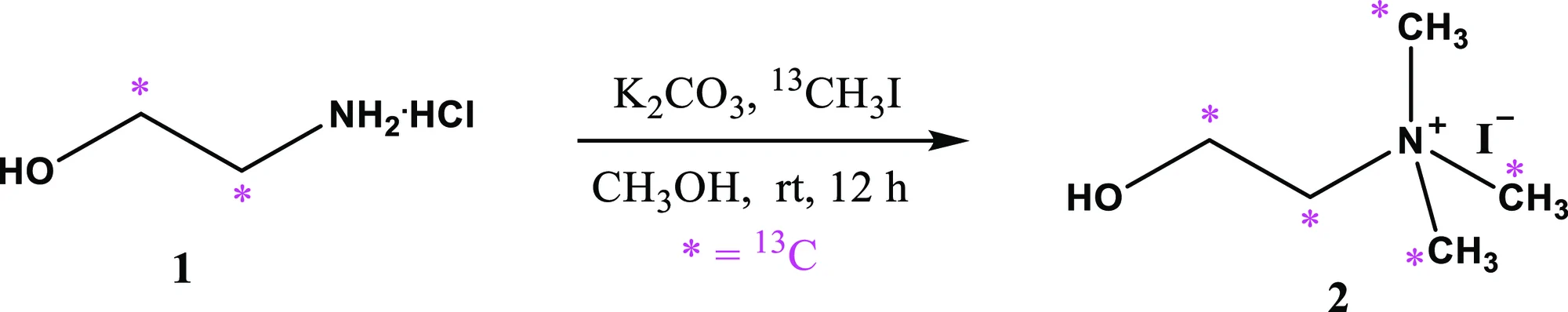
Synthesis of U–^13^C-Choline Iodide (**2**)

### Murine Primary Hepatocyte Isolation

The whole procedure was deliberated and accepted by Seoul National University Institutional Animal Care and Use Committee (IACUC; assurance number SNU-260223–8). C57BL/6 mice (male, 6 weeks old) were purchased from Orient Bio Laboratory Animal Research Center Co., Ltd. (Seoul, Korea). Mice were kept (*n* = 5 per cage) for more than a week to acclimatize before carrying out the experiments. Primary hepatocytes were isolated from mouse livers using a protocol described by Charni-Natan and Goldstein.^22^ Briefly, mice were anesthetized, and the inferior vena cava was cannulated to initiate liver perfusion using a 24G catheter (Korea Vaccine Co., Ltd., Ansan, Korea) and a peristaltic pump (Cat# MP2000, Tokyo Rikakikai Co., Ltd., Tokyo, Japan). The liver was perfused with an EDTA (Cat#03690, Sigma-Aldrich, St. Louis, MO, USA)-containing DPBS (Cat# LB001-02, WELGENE Inc., Gyeongsan, Korea) and then 0.4 mg/mL of collagenase (Cat# C9891, Sigma-Aldrich, St. Louis, MO, USA)-containing DMEM. After digesting the liver with collagenase-containing media, the liver was excised and gently dissociated to release hepatocytes in DMEM. The hepatocyte suspension was filtered through a 70 μm cell strainer (Cat# 352350, Corning, Corning, NY, USA) and centrifuged (50 × g, 3 min). The supernatant was removed, and hepatocytes were washed with DMEM, and this washing step was done three times. Cells were plated on collagen-coated dishes with DMEM (Cat# LM001-05, WELGENE Inc.) supplemented with 10% fetal bovine serum (FBS) (Cat# S001-07, WELGENE Inc.) and 1% penicillin–streptomycin (Cat# LS202-02, WELGENE Inc.) and incubated at 37 °C with 5% CO_2_ in a humidified incubator. The media was replaced after 6 h and incubated overnight.

### Cell Culture and Isotope Labeling

Human nontransformed hepatocyte cell lines (NKNT-3 and HL-7702) and human hepatoma cell lines (Hep3B) were cultured in DMEM, supplemented with 10% FBS and 1% penicillin–streptomycin, and incubated at 37 °C with 5% CO_2_ in a humidified incubator. Cell lines were routinely tested for mycoplasma contamination using Myco-Read Mycoplasma Detection Kit (Cat# SMD0173, Biomax Ltd., Guri, Korea). For isotope labeling, cells were seeded in 150 mm plates and maintained overnight before isotope labeling. The culture medium was replaced with a fresh medium containing 0.2 mM U–^13^C-Cho, 0.2 mM U–^13^C-Etha (Cat# CLM-274-PK, Cambridge Isotope Laboratories, Inc., Tewksbury, MA, USA), 10% dialyzed FBS, and 1% penicillin–streptomycin and incubated at 37 °C with 5% CO_2_ in a humidified incubator for 3 days before metabolite extraction.

### Plasmids, Lentiviral Production, and Transduction

For overexpressing PEMT, lentiviral vectors expressing PEMT (Cat# VB900133–9015kbx) and control (VB010000–9389rbj) were designed and obtained from VectorBuilder (Chicago, IL, USA). Lentiviral vectors were transfected into HEK293T cells with psPAX2 and pMD2G using Lipofectamine 2000 (Cat#11668019, Thermo Fisher Scientific Inc., Waltham, MA, USA). Lentiviral solutions were harvested after 48 and 72 h and concentrated with a Lenti-X-Concentrator (Cat# 631331, Takara Bio Inc., Shiga, Japan). For viral transduction, Hep3B cells were incubated with a concentrated lentiviral solution with a 10 μg/mL Polybrene (Cat# TR-1003, Sigma-Aldrich) for 24 h, followed by antibiotic selection (puromycin, Cat# P8833, Sigma-Aldrich) for 4–6 days. Overexpression efficiency was measured by Western blot analysis.

### Metabolite Extraction

Cells were washed with cold PBS, scraped into 1.5 mL of cold PBS, and transferred into microcentrifuge tubes. Cells were pelleted by centrifugation and washed with cold PBS. After discarding the supernatant, the pellet was dissolved in 400 μL of MeOH and 200 μL of CHCl_3_ and vortexed. An additional 200 μL CHCl_3_ and 200 μL distilled water were added, vortexed, and centrifuged at 15,800 × g at 4 °C for 20 min. The separated upper and lower phases were transferred into each new microcentrifuge tube, dried under a vacuum evaporator, and stored at −80 °C until further analysis.

### NMR Method

The extracted lower phase was dissolved in 500 μL of CDCl_3_ and transferred to Wilmad-LabGlass High-Throughput NMR Tubes (Cat# WG-1000–7, Wilmad-LabGlass, Vineland, NJ, USA). NMR experiments were performed on a Bruker Avance III HD 800 MHz NMR spectrometer (Bruker Biospin GmbH, Rheinstetten, Germany) equipped with a 5 mm triple resonance inverse (TCI) Cryoprobe at 298 K at the College of Pharmacy, Seoul National University (Seoul, Korea). The modified 2D ^1^H–^13^C-HMBC spectra were acquired with the parameters as follows:

Spectral width (SW): 4.7158 ppm along the ^13^C and 5.9928 ppm along the ^1^H dimensions; O1P = 3.430 ppm, O2P = 66.173 ppm; time domain (TD): 2396 (for a direct dimension) and 96 (for an indirect dimension); number of scan (NS) = 32; acquisition time: 250 ms; for each time point; and an inter scan delay (d1) = 281 ms (see Supporting Information for detailed pulse sequence parameters).

NMR data were processed and analyzed by using TopSpin 4.2.0 (Bruker). For quantification, integration values of ^1^H–^13^C were corrected with a coefficient value of 1.092.

### Western Blot Analysis

Western blot analysis was performed as described before.^23^ Briefly, samples were lysed and quantified using the BCA Protein Assay Kit (Thermo Fisher Scientific, Waltham, MA, USA). Quantified samples were loaded onto 13% SDS-PAGE gels and underwent electrophoresis. Separated proteins were transferred to a nitrocellulose membrane, blocked with 5% BSA in TBST for 1 h at room temperature with mild shaking, and incubated with primary antibodies at 4 °C overnight. Primary antibodies of β-actin (Cat# sc-47778, Santa Cruz Biotechnology, Inc., Dallas, TX, USA) and PEMT (Cat# E-AB-52055, Elabscience, Houston, TX, USA) were used for Western blot analysis. Goat antimouse IgG (H + L) Secondary Antibody, HRP (Cat# 31430, Invitrogen, Thermo Fisher Scientific), and goat antirabbit IgG (H + L) Secondary Antibody, HRP (Cat# 31460, Invitrogen, Thermo Fisher Scientific), were used for secondary antibody. Bands were detected with a chemiluminescent reagent (West Save Star, Cat# LF-QC0106, Abfrontier, Seoul, Korea) and Image Analyzer (SOLO.6x)-Chemi DOC (VILBER LOURMAT, Collégien, France).

### Statistical Analysis

Experiments were conducted in three replicates, unless indicated otherwise, and the results of the replicates were drawn to graph with mean value and standard deviation using GraphPad Prism (ver. 9.3.0). The statistical significance was analyzed with ordinary one-way ANOVA and unpaired Student’s *t*-test (“*”: *p* < 0.05; “**”: 0.001 < *p* ≤ 0.01; “***”: 0.0001 < *p* ≤ 0.001; “****”: *p* ≤ 0.0001; and “ns”: ‘not significant’).

## Results and Discussion

### Overall Strategy and Necessary Experimental Steps for the Distinction of PEMT and Kennedy Pathways

For an efficient way of discriminating between PEMT and Kennedy pathways for PtdCho biosynthesis in a single experiment, we devised a strategy using U–^13^C-Etha and U–^13^C-Cho as the precursors for the respective pathways (Figure 1A). Specifically, PtdCho synthesized from U–^13^C-Etha through PEMT contains unlabeled methyl groups originating from SAM during the methyl transferase process, whereas the one from U–^13^C-Cho through the Kennedy pathway carries the uniformly labeled Cho moiety. As the products are different only in terms of ^12^C- vs ^13^C-labeled status in the *N*-methyl carbon, the two pathways can be compared in one experiment as long as the ^12^C vs ^13^C ratio of the methyl carbons can be determined. Although the idea itself seemed simple initially, the actual implementation required the chemical synthesis of U–^13^C-Cho due to the commercial unavailability and optimization of the existing HMBC pulse sequence for compensation of the NMR property difference between the isotopically different species.

**Figure 1:**
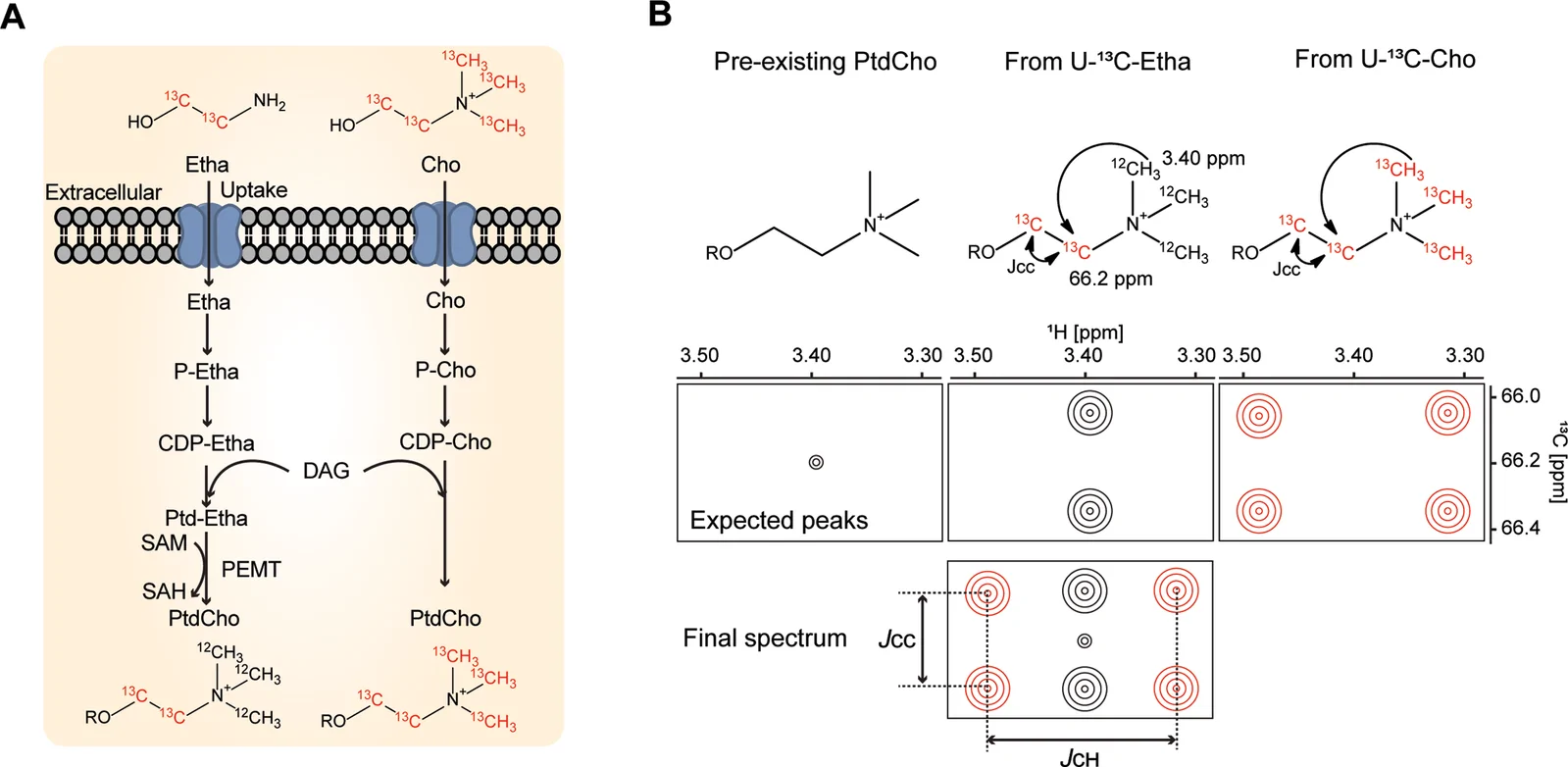
Development of the labeled tracing strategy to measure the PtdCho from Cho and Etha. (A) Overall scheme of PtdCho synthesis from U–^13^C-Cho and U–^13^C-Etha via PEMT or Kennedy pathway. (B) Theoretic spectra of PtdCho from pre-existing, U–^13^C-Etha, U–^13^C-Cho, or a mixture of them.

First, as for the synthesis of U–^13^C-Cho, the use of unlabeled Etha and ^13^C-methyl labeled Cho might be an alternative (with their CH_2_ in natural abundance ^13^C), but it did not give a measurable signal on the HMBC experiment on cell extracts (data not shown), and we had to use the uniformly ^13^C labeled Cho and Etha for sensitivity reasons. U–^13^C-Cho was synthesized through a straightforward substitution reaction involving U–^13^C-Etha and U–^13^C-methyl iodide. As we also used U–^13^C-Etha for the PEMT pathway substrate, U–^13^C-methyl iodide was the only additional reactant for the synthesis. Second, with the distinction between ^12^C- vs ^13^C-labeled methyl carbons, we employed the HMBC experiment since it does not depend on the ^13^C status of the methyl carbon for NMR peak generation but correlates the methyl proton signals to the 1-CH_2_ carbon with a three-bond coupling. Theoretically, a simple one-dimensional proton-NMR exploiting the ^13^C satellite peaks could provide the desired information, but extensive peak overlaps preclude its usage in this application. HMBC, with its much higher practical resolution provided by the second dimension, can generate the ^12^C- and ^13^C-labeled methyl signals without any overlap, as long as the broadband carbon decoupling is turned off during the direct acquisition, as is usually the case. As schematically described in Figure 1B, the single HMBC spectra will contain the center peak (in black) from the ^12^C methyl carbon through the PEMT pathway and the side peaks (in red), split by the ^1^ *J*_CH_ from the ^13^C methyl carbon, from the Kennedy pathway. The figure features additional splitting of the peaks in the indirect carbon dimension due to our use of U–^13^C-Etha and U–^13^C-Cho as the precursors. Since both 1-CH_2_ and 2-CH_2_ carbons in PtdCho are ^13^C isotope-labeled regardless of the biosynthetic pathway, the ^13^C–^13^C *J*-coupling interaction between them (*J*_12_) splits the 1-CH_2_ correlation signal into a doublet along the indirect carbon dimension appearing at Ω_c_ ± *J*_12_/2, with the Ω_c_ being its carbon chemical shift. As a result, the final pattern will be two peaks along the carbon dimension centered at Ω_c_ = 66.2 ppm for the PEMT and four peaks along both dimensions centered at Ω_H_ = 3.40 ppm and Ω_c_ = 66.2 ppm for the Kennedy pathway. The merit of this splitting is that the signal from pre-existing PtdCho appears as a singlet in both *F*_*1*_ and *F*_*2*_ dimensions, allowing for more accurate comparison of the pathway activities involving the added labeled precursors.

### Modification of the HMBC Pulse Sequence to Compensate for the Isotopic Difference

Due to the NMR property differences between the ^12^CH_3_ and ^13^CH_3_ groups in the PtdCho in each pathway, it was necessary to modify the HMBC pulse sequence if we were to perform any quantitative comparison between PtdCho generated by the two pathways. First, unlike N–^12^CH_3_–PtdCho whose interaction with *N*-methyl carbon can be neglected due to the low natural abundance of ^13^C, in the case of N–^13^CH_3_–PtdCho, N–^13^CH_3_ proton has three additional ^1^H–^13^C *J*-coupling interactions, other than the desired 3-bond ^1^H–^13^C *J*-coupling to the 1-CH_2_ (shown in black; Figure 2A); one with the directly attached methyl ^13^C carbon (one-bond coupling), and two others with the other ^13^C methyl carbons (three-bond coupling, shown in blue; Figure 2A). With a conventional HMBC method, these interactions inhibit the complete polarization transfer from N–^13^CH_3_ proton to the 1-^13^CH_2_ carbon, resulting in a reduction of the HMBC signal magnitude compared to that in natural abundance N–^12^CH_3_ as in PEMT-generated PtdCho. To address this, band-selective insensitive nuclei enhanced by polarization transfer (INEPT) using an inversion pulse (red dotted rectangle; Figure 2B), its central frequency and effective bandwidth matched to the 1-^13^CH_2_, has been employed instead of conventional nonselective long-range ^1^H–^13^C polarization transfer sequence,^24^ thus enabling a 100% polarization transfer from both N–^12^CH_3_ and N–^13^CH_3_ protons to the 1-^13^CH_2_ carbon, simultaneously. Second, during the delay for the coherence selection gradient, ^13^C–^13^C *J*-coupling interaction between ^13^C_1_ and ^13^C_2_ evolves, generating phase distortion hampering accurate signal analysis.^25^ This phase distortion was suppressed by adding the reversed clean in-phase (CLIP) INEPT sequence^26^ involving a selective refocusing pulse on the ^13^C_1_ carbon. This prevents the generation of antiphase signals, which typically have small coupling magnitudes and opposite signs, eliminating signal cancellation due to overlap between signals with different phases and ensuring the generation of an absorptive signal (*see the* Supporting Information *in detailed analysis*). Third, the relaxation constants (T_1_ and T_2_) are different between N–^12^CH_3_ and N–^13^CH_3_ because N–^13^CH_3_ has a dipole–dipole interaction between the proton and NMR-active ^13^C. This interaction is the major source of T_1_ relaxation and a significant source of T_2_ relaxation. As signal intensity is affected by these relaxation parameters, we needed to consider the difference between N–^12^CH_3_ and N–^13^CH_3_–PtdCho. Additionally, typical small molecule NMR experiments use a smaller interscan delay than required for the complete relaxation (typically, >5 times of T_1_) to enhance signal-to-noise ratio (SNR) in a given time. In this case, there will be an imbalance in the nuclear spin population of the two kinds of N–CH_3_ protons at each scan, leading to cumulative errors in the final HMBC signals. Therefore, with the number of scans (NS) and the F1/F2 time-domain (TD) sizes fixed, we optimized the excitation angle together with the interscan delay d1, the acquisition time, and the transfer delay τ by maximizing SNR/√T (T being the total acquisition time) using a simulated-annealing search over a Bloch-equation signal model, which yielded d1 = 281 ms. Because the N–^12^CH_3_ and N–^13^CH_3_ signals overlap in the cell extract, their species-specific T1 and T2 cannot be measured directly there; we therefore determined them on the pure isotopologue standards (Table S1) and propagated these measured values through the full multiscan acquisition by Bloch-equation simulation. Then, with the parameters and experimentally measured T_1_ and T_2_ values for each N–^12^CH_3_ and N–^13^CH_3_–PtdCho, we computed, under identical acquisition conditions for all species, the ratio of the cumulative signals and obtained a correction factor of 1.092 between the two molecules (*see the* Supporting Information *in detail*). This value was applied to the NMR signals to calculate the final ratio between the PEMT and Kennedy pathway.

**Figure 2:**
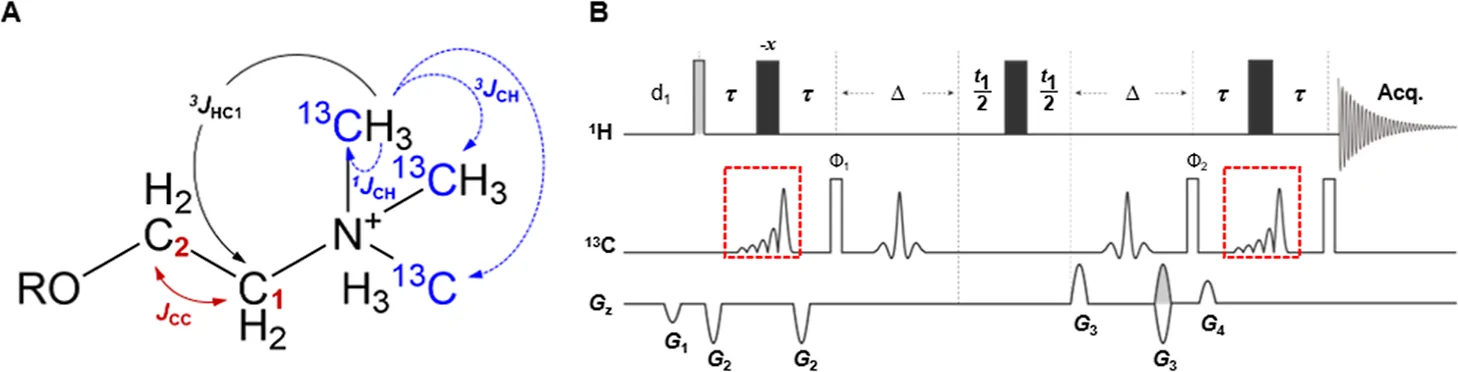
NMR spectroscopy for the optimal HMBC signal acquisition. (A) Schematic illustration of coupling pathways contributing to HMBC signals, highlighting J_CH_-mediated one-bond and long-range correlations and additional ^13^C–^13^C coupling-induced splitting. (B) Proposed pulse sequence of a modified 2D ^1^H–^13^C HMBC. The narrow gray bar denotes a 1.08 × 90° hard pulse. Narrow white and wide black bars represent 90° and 180° hard pulses, respectively. Asymmetric and symmetric waves in the ^13^C channel indicate selective inversion and selective refocusing pulses for the ^13^C_1_-[N–CH_2_] of PtdCho, respectively. For the selective inversion, the “Iburp2” waveform was employed with an 18.1 ms duration, and for the selective refocusing, the “Rsnob” waveform was employed with a 2.33 ms duration. The interscan delay (d1) and acquisition time (Acq.) were set to 281 and 250 ms, respectively. The transfer delay τ was set to 21.44 ms, obtained—together with d1 and AQ—from the SNR/√T optimization (Supporting Information); it is not 1/(4·^3^J_CH_), as τ was shortened relative to the nominal transfer condition to limit T_2_ losses. The measured long-range coupling is ^3^ *J*_HC1_ ≈ 7 Hz.; Δ, constant delay for the coherence-selection gradient pair, with the selective ^13^C1 refocusing pulse at its center. All pulses are of phase x, unless otherwise indicated. The phase cycling is as follows. Φ_1_ = *x*, -*x*; Φ_2_ = *x*, *x*, -*x*, -*x*; Φ_rec_ = *x*, -*x*, -*x*, *x*. Gradient ratios: G_3_/G_4_ = 80:40.2. The second G_3_ gradient consists of a bipolar gradient pair (depicted in gray/white) for the echo–antiecho detection mode. G_1_ and G_2_ are crusher and homospoil gradient pulses, respectively.

### Apply Selective HMBC for Measuring PEMT Activity in Various Liver Cells

With the strategy and the pulse sequence set up, we applied them to the actual measurement of the Kennedy and PEMT pathways for PtdCho synthesis. We employed various liver cells (murine primary hepatocytes (Pri-Hep), human nontransformed hepatocyte cells (HL-7702 and NKNT-3), and human hepatoma cells (Hep3B)) because the liver is a major organ for PtdCho synthesis and both pathways are active in the liver.^5^ We treated U–^13^C-Etha and U–^13^C-Cho simultaneously to the cells, followed by two-phase extraction. The organic phase, which predominantly contains PtdCho while excluding water-soluble precursors such as Cho, Etha, and CDP-Cho, was subjected to the modified HMBC analysis. The results clearly show that the expected peak patterns, i.e., center peaks from PEMT and side peaks from Kennedy pathways, appeared and that the peaks are split according to the isotope labeling (Figure 3A). Specifically, we were able to observe the splitting by the coupling between N- and O-methylene groups (*J*_CC_ = 40.0 Hz) and the proton–carbon coupling in N–^13^C-methyl groups (*J*_CH_ = 140.0 Hz) (Figure 3A; the full 2D HMBC spectrum is provided in the Supporting Information). The high sensitivity of our proton-based HMBC approach, compared to the previous ^13^C-based one,^16^ allowed us to obtain data in just 30 min. Notably, the one-dimensional projection of the HMBC spectrum of this region from murine primary hepatocyte exhibited significant difference from the simple one-dimensional ^1^H-spectrum of the same sample (Figure 3B). The much larger peak from the latter approach is either due to preexisting unlabeled PtdCho or overlap with unrelated peaks, showing the necessity of our approach for the proper estimation of the two pathways. Based on the ^13^C/^12^C ratio calculation as described above, murine primary hepatocytes and human liver-origin cell lines exhibited differential contributions from the two pathways to the PtdCho synthesis (Figure 3C). Specifically, the PEMT and Kennedy pathways showed similar activities for the PtdCho synthesis in murine primary hepatocytes, with the PEMT pathway contributing up to 47% of the total PtdCho synthesis. This result is rather higher than the previous literature for rats or mice, where PEMT was reported to account for 10–30% of hepatic PtdCho de novo synthesis.^4,5,16^ This discrepancy could be due to the different experimental conditions, such as incubation time or species. In contrast to murine primary hepatocytes, human liver-origin cell lines showed a much higher dependence on the Kennedy pathway, contributing up to 90%, regardless of their cancer (Hep3B) or normal cell line (HL-7702 and NKNT-3) status. To our knowledge, no research has directly compared the contribution of PtdCho synthesis pathways in human liver-origin cells. In addition, it will be next to impossible to ethically obtain human normal liver tissue samples for PtdCho analysis after in vivo isotope labeling. For this reason, Pynn et al. extrapolated the results from the relationship between mouse liver and plasma PtdCho to estimate the human liver PtdCho synthesis from human plasma PtdCho data.^4^ The results predicted that the PEMT pathway will contribute about 0.53%/h to normal human liver PtdCho synthesis, which is about ∼10 times lower than the PEMT pathway flux in mouse liver (5.04%/h) measured experimentally in the same study. This much lower PEMT activity seems consistent with our results showing about 5 times lower PEMT activity in human cells than mouse primary cells. Still, it is important to consider another possibility, considering that our normal liver cells are immortalized cell lines. Therefore, the observed decrease in the PEMT pathway contribution in human cells might be due to the acquisition of immortality or rapid cell division. In addition, despite the previously suggested similarity between mouse and human liver PtdCho synthesis, it still cannot be excluded that this difference might be simply due to the species difference. Further studies are warranted to get deeper insights.

**Figure 3:**
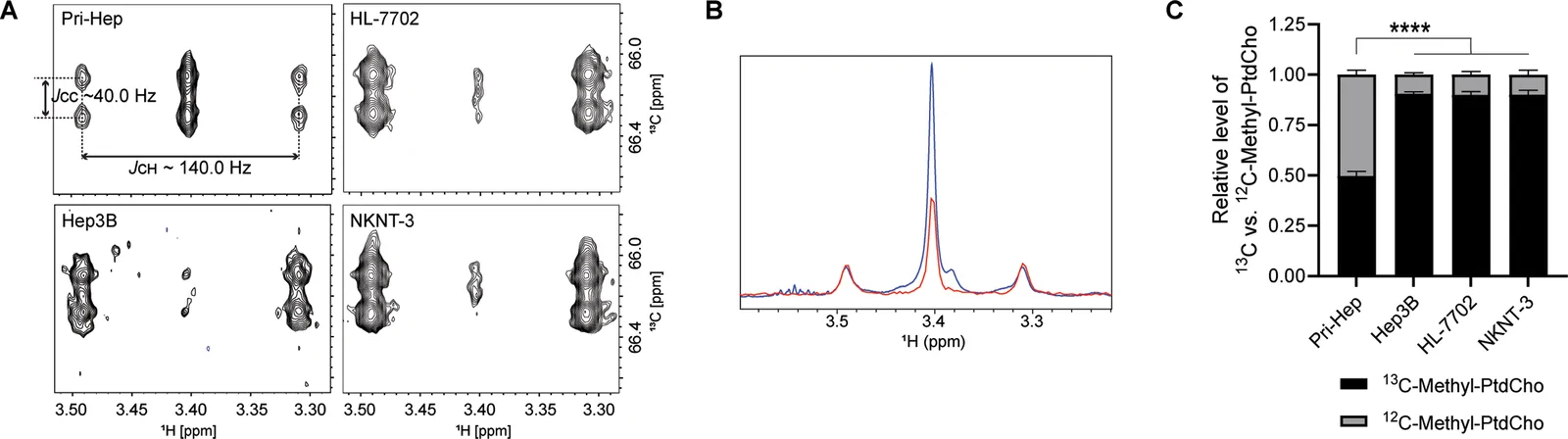
PtdCho level and contribution of each pathway in various liver cells. (A) The HMBC spectra showing the correlation between protons of *N*-methyl (3.40 ppm) and the 13-carbon of *N*-methylene (66.2 ppm). (B) Overlayed spectra of PtdCho from 1D-HMBC (red) and 1D-proton (blue) of primary hepatocyte. (C) Ratio of ^13^C/^12^C of methyl groups in liver cells. Data were analyzed using ordinary one-way ANOVA, and a post hoc two-stage step-up method of Benjamini, Krieger, and Yekutieli was applied for multiple comparisons.

In addition to the relative contribution of each pathway in a particular cell, our method also enabled us to compare the bulk activity of one pathway across different cell types. For example, both the Kennedy pathway and PEMT pathway showed more than two times higher activities in human nontransformed hepatocyte cell lines (HL-7702 and NKNT-3) than human hepatoma cell (Hep3B) (Figure S1A,B). We also measured the PEMT expression level in the cells using Western blotting (Figure S1C), which matched quite well with the above bulk PEMT activity. This suggests that PEMT is the rate-limiting step of the PtdCho synthesis from Etha and that PEMT activity is mostly controlled by the enzyme amount itself rather than other mechanisms such as post-translational modification or protein–protein interaction. Interestingly, the sum of the PEMT and Kennedy bulk activity, representing the total PtdCho synthesis, was lower in Hep3B liver cancer cells than in other cells, which might suggest that human liver cancer cells could be less dependent on de novo PtdCho synthesis pathways (Figure S1D). This could be due to liver cancer heterogeneity, as other studies have reported no significant difference in total PtdCho levels between liver cancer and adjacent normal liver tissue.^18,27,28^ The discrepancy may also result from differences in the experimental settings. In our study, we used a ^13^C-labeled substrate for NMR, which reflects the newly synthesized amount of total PtdCho, whereas other studies measured either a combination of individual detected PtdCho species using mass spectrometry^18,27^ or the total existing amount of PtdCho using thin layer chromatography.^28^ As we used only one cancer cell line, it will be interesting to see whether similar results are observed in other live cancer cells with our method and other modalities.

### Effect of Overexpression of PEMT in a Human Cancer Cell Line

There have been several lines of evidence for the tumor suppressor roles of PEMT using rat liver cancer cells.^10–12^ In addition, an observational human study reported lower PEMT expression in human liver cancer than in adjacent normal liver tissue,^29^ which is consistent with our result. Therefore, we tested the hypothesis that PEMT overexpression in human cancer cells will increase the PEMT contribution to the PtdCho synthesis with the concomitant suppression of tumor cell proliferation. Lentiviral transduction of the PEMT gene into the Hep3B human liver cancer cells with very little basal expression of PEMT highly increased its expression (Figure 4A). Then, we applied our HMBC NMR approach to the cells to estimate the contributions of PEMT and Kennedy pathways (Figure 4B). Based on the ^13^C/^12^C ratio calculation using the obtained spectra, the PEMT contribution to the PtdCho synthesis was greatly increased to about 50%, matching that of the mouse primary liver hepatocytes measured above (Figure 4C). Further detailed analysis showed that the PtdCho amount synthesized by the PEMT pathway was about 3.2 times higher than that in control cells, while the PtdCho synthesized by the Kennedy pathway slightly decreased in PEMT OE cells (Figure S2A,B). These results confirm half of our hypothesis and also validate our analytical approach. To test the other half of the hypothesis, tumor suppressor roles of PEMT, we measured cell proliferation and tumorigenicity using the sulforhodamine B (SRB) assay and colony formation assay, respectively. Surprisingly, the results showed that PEMT overexpression did not induce any changes in proliferation or tumorigenicity (Figure S3A,B). This result shows that PEMT may not be a tumor suppressor gene in human liver cancer, even though it does modulate PtdCho synthesis. It should be noted that PEMT’s tumor suppressor roles in the liver have been around for long but are based on rat liver studies. Therefore, species differences may need to be considered in extrapolating PEMT’s functions, which is also evident in the large difference in PEMT’s contribution to PtdCho synthesis according to species as stated above (see Figure 3 and ref 4). Another possible explanation is the presence of different PEMT isoforms. In our study, we used the human PEMT long isoform (PEMT-L), while other studies mainly employed the rat PEMT2 or human short isoform (PEMT-S).^10,15,30^ Given that PEMT-L and PEMT-S have distinct substrate specificities—PEMT-L preferentially acts on ether-linked PtdCho species with shorter chains, whereas PEMT-S favors PtdCho and ether-linked PtdCho species with longer chains—this difference may account for the varying phenotypes observed.^31^

**Figure 4:**
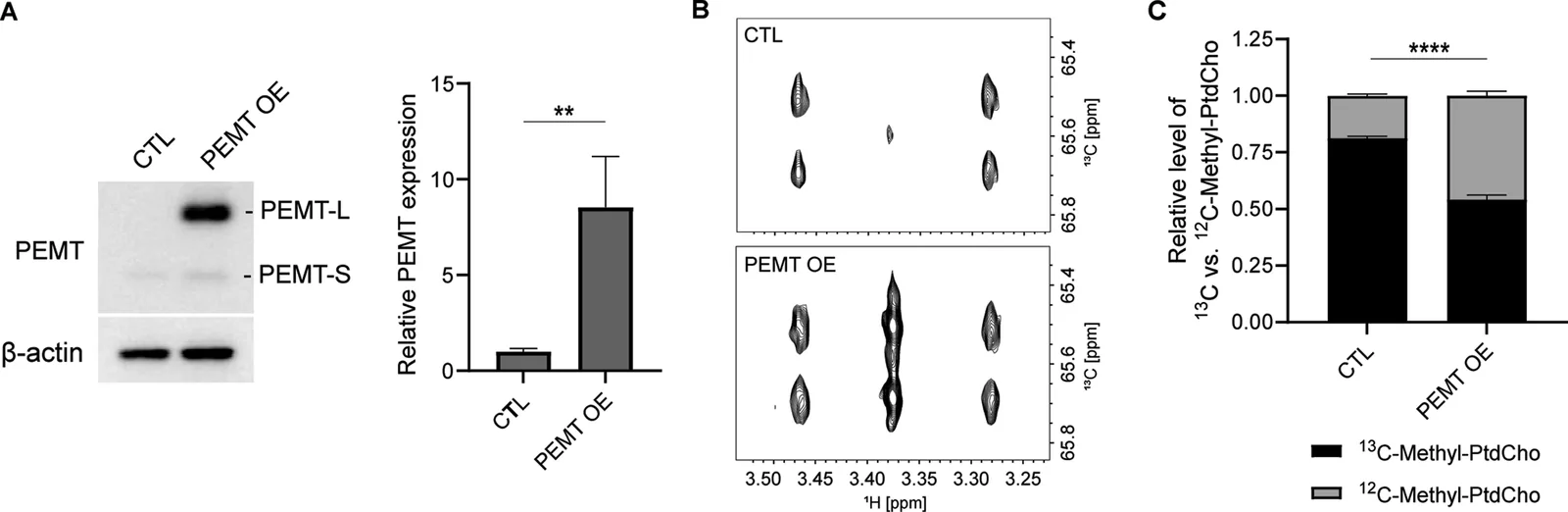
Changes in the PtdCho synthesis pathway in PEMT overexpressed Hep3B cells. (A) Western blot analysis of PEMT level in Hep3B control (CTL) and PEMT overexpressed (PEMT OE) cells and its quantification graph was analyzed by unpaired *t*-test. (B) The HMBC spectra showing the correlation between protons of *N*-methyl (3.40 ppm) and 13-carbon of *N*-methylene (66.2 ppm). (C) Ratio of ^13^C/^12^C of methyl groups in Hep3B CTL and PEMT OE cells. Data were analyzed by unpaired *t*-test.

The previous study labeled PtdCho using 1-^13^C-Cho and 2-^13^C-Etha based on ^13^C NMR exhibits the severe overlapping of unlabeled PtdCho due to the low labeled fraction in vivo.^16^ Our approach again leverages the high resolution of the HMBC sequence to distinguish the ^13^C natural abundance of unlabeled PtdCho. In addition, the difference in relaxation time in C1 and C2 of the choline moiety of PtdCho should require a very long delay time to obtain the correct estimation of PtdCho.

There is one theoretical concern for our approach using ^13^C-Cho and ^13^C-Etha as the tracer for Kennedy and PEMT pathways, respectively. Since the ^13^C methyl of ^13^C-Cho can be incorporated into SAM through an array of enzymes, and the SAM eventually methylates PtdEtha to produce PtdCho through PEMT, there is a possibility of the ^13^C-methyl group incorporation into PtdCho through PEMT (we call this choline–PEMT pathway). Therefore, the ^13^C satellite peak might include some contribution from the Cho-PEMT pathway. However, under a general setup with physiological choline concentration (∼200 μM in our case), the ^13^C isotopic enrichment of SAM was very low in all human liver cell lines examined, including both normal human liver cell lines and human liver cancer cell lines (approximately 0.4–0.5% after excluding the natural abundance contribution of 13.17%), indicating that the Cho-PEMT contribution to the total PEMT-generated PtdCho will be even smaller (Figure S4). Therefore, the possible error of PEMT contribution will be much smaller than the ^13^C enrichment of SAM in human liver cell lines. In contrast, the ^13^C isotopic enrichment of SAM in primary hepatocytes was ∼9% (Figure S4), which is expected since BHMT expression is much higher in primary hepatocytes than in human liver cell lines (data not shown). Therefore, the potential overestimation of the Kennedy pathway contribution to PtdCho synthesis in primary hepatocytes in our system would be expected to remain below 9%. This estimated error range is consistent with a previous study performed under another physiological condition, in which the D_3_ enrichment of PtdCho (representing the Cho-PEMT contribution to total PtdCho, shown in Figure 3 of ref 4) was less than 5% in mouse samples and less than 1% in human samples.^4^ Overall, the contribution of the Cho-PEMT pathway (^13^C enrichment of PtdCho through PEMT) to total PtdCho in our experimental setting is expected to be minimal and thus should not be concerning.

## Conclusions

In summary, we developed a high-sensitivity, modified HMBC NMR-based tracer strategy that enables simultaneous and quantitative discrimination of the PEMT and Kennedy pathways for phosphatidylcholine biosynthesis in a single experiment. Application of this method revealed clear species-specific differences, with murine primary hepatocytes exhibiting comparable contributions from both pathways, whereas human liver-derived cells relied predominantly on the Kennedy pathway. PEMT activity closely reflected enzyme expression levels, supporting PEMT abundance as a key determinant of ethanolamine-derived PtdCho synthesis. This HMBC-based platform provides a robust and broadly applicable tool for quantifying parallel lipid biosynthetic fluxes and can be extended to investigate metabolic reprogramming in liver physiology, cancer, and other lipid-associated diseases.

## Supplementary Material



## Notes

Materials; T1 and T2 relaxation measurements; T1 measurement reports of [N–^13^CH_3_]­PtdCho and [N–^12^CH_3_]­PtdCho; T2 measurement reports of [N–^13^CH_3_]­PtdCho and [N–^12^CH_3_]­PtdCho; necessity of band-selective INEPT for quantitative HMBC of [N–^13^CH_3_]­PtdCho; product–operator description of the modified HMBC sequence; quantitative correction of ^13^C-isotope-dependent relaxation effects in HMBC spectroscopy; Python code for the correction factor calculation; Kennedy pathway activity and PEMT pathway activity in various liver cells; Kennedy pathway activity and PEMT pathway activity in CTL and PEMT OE cell; proliferation of CTL and PEMT OE Hep3B cells; effect of choline–PEMT contribution to total PEMT-generated PtdCho; the ^1^H NMR spectrum of 2 in CD_3_OD (400 MHz); the ^1^H NMR spectrum of 2 in D_2_O (400 MHz); the mass spectrum of 2; the ^13^C NMR spectrum of 2 in CD_3_OD (100 MHz); the ^1^H–^1^H COSY NMR spectrum of 2 (400 MHz); the ^1^H–^13^C HSQC NMR spectrum of 2 (400 MHz); the ^1^H–^13^C HMBC NMR spectrum of 2 (400 MHz); NMR assignment of 2; original Western blots for Figure S1C and Figure 4A; and full 2D HMBC spectra corresponding to Figure 3A and peak assignment (PDF)
