# Supplementary material for: Quantitation of PEMT and Kennedy Pathways in Liver Phosphatidylcholine Biosynthesis Using NMR
Source: Anal Chem. 2026 Aug 29;98(36):26503–11. doi: 10.1021/acs.analchem.6c02190 (PMC13584693; doi:10.1021/acs.analchem.6c02190)
Supplement: Supplementary file 1 [file ac6c02190_si_001.pdf]

Supporting Information for: Quantitation of PEMT and Kennedy Pathways in Liver Phosphatidylcholine Biosynthesis Using NMR

Ji Yun Lee[1,‡], Tin Tin Manh Nguyen [1,‡], Thi Ha Nguyen [1], Eunbyul Cho[1], Seungjoon Lee[1], Hyuk Nam Kwon[2], Lak Shin Jeong[3], Dnyandev B. Jarhad[3], Jin Wook Cha[4,], Sunghyouk Park[1,**]*

[1] Natural Products Research Institute, College of Pharmacy, Seoul National University, Seoul 08826, Republic of Korea.

[2] Department of Biological Sciences, University of Ulsan, Ulsan, 44610, Republic of Korea.

[3] Research Institute of Pharmaceutical Sciences, College of Pharmacy, Seoul National University, Seoul 08826, Republic of Korea; Future Medicine Co., Ltd, 54 Changup-ro, Sujeong-gu, Seongnam, Gyeonggi-do 13449, Republic of Korea.

[4] KIST Gangneung Institute of Natural Products, Natural Product Drug Development Division, Center for Natural Product Systems Biology, Gangneung 25451, Republic of Korea.

[‡] These authors contributed equally to this work

[*] Correspondence: elmarit@kist.re.kr

[**] Correspondence: psh@snu.ac.kr

Table of Contents

Materials……………………………………………………………………………………...….S3

T1 and T2 relaxation measurements……………..…………………………………………S4

T1 measurement reports of [N-$^{13}CH_3$]PtdCho and [N-$^{12}CH_3$]PtdCho…………………...……S4

T2 measurement reports of [N-$^{13}CH_3$]PtdCho and [N-$^{12}CH_3$]PtdCho……………….….….……S8

Necessity of band-selective INEPT for quantitative HMBC of [N-$^{13}CH_3$]PtdCho………….S12

Product-operator description of the modified HMBC sequence……………………….….S16

Quantitative correction of ^{13}C-isotope-dependent relaxation effects in HMBC spectroscopy..S18

Table S1: T1 and T2 relaxation constants for [N-$^{12}CH_3$]PtdCho and [N-$^{13}CH_3$]PtdCho…….S19

Python code for the correction factor calculation……………………..……………...……S22

Figure S1: Kennedy pathway activity and PEMT pathway activity in various liver cells...……………………………………………………………………………………...S33

Figure S2: Kennedy pathway activity and PEMT pathway activity in CTL and PEMT OE cell…………………………………………………………………….……………………...S34

Figure S3: Proliferation of CTL and PEMT OE Hep3B cells…………….………..…………S35

Figure S4: Effect of choline-PEMT contribution to total PEMT-generated PtdCho…………S35

Figure S5: The ^{1}H NMR spectrum of 2 in CD_3OD (400 MHz)…………………….…...……S36

Figure S6: The ^{1}H NMR spectrum of 2 in D_2O (400 MHz)………………………..………S37

Figure S7: The mass spectrum of 2……………………………………………….………..………S38

Figure S8: The ^{13}C NMR spectrum of 2 in CD_3OD (100 MHz)…………………...…………S39

Figure S9: The ^{1}H-^{1}H COSY NMR spectrum of 2 (400 MHz)……………….………………S40

Figure S10: The ^{1}H-^{13}C HSQC NMR spectrum of 2 (400 MHz)…………………...…..…..…S41

Figure S11: The ^{1}H-^{13}C HMBC NMR spectrum of 2 (400 MHz)………………………….……S42

Table S2: NMR assignment of 2…………………………………………………………...S43

Figure S12: Original western blots for Figure S1C…………………………………..……S44

Figure S13: Original western blots for Figure 4A……………………………………………S44

Figure S14: Full 2D HMBC spectrum corresponding to Figure 3A (Pri-Hep)…………..…...S45

Figure S15: Full 2D HMBC spectrum corresponding to Figure 3A (HL-7702)……….……..S46

Figure S16: Full 2D HMBC spectrum corresponding to Figure 3A (Hep3B)..………..……...S47

Figure S17: Full 2D HMBC spectrum corresponding to Figure 3A (NKNT-3)……….……...S48

Table S3: HMBC peak assignment……………………..…………………….….…………S48

Reference……………...……………..…………………………….……..........……..…S49

Materials

DL-α-Phosphatidylcholine, dipalmitoyl (U-^{13}C, 98%) was purchased from Cambridge Isotope Laboratories, Inc. (MA, USA), and 1,2-dipalmitoyl-rac-glycero-3-phosphocholine was obtained

from Sigma-Aldrich (MO, USA). These compounds were used as standards for T1 and T2 relaxation measurements of [N-$^{13}CH_3$]PtdCho and [N-$^{12}CH_3$]PtdCho, respectively.

T1 and T2 relaxation measurements

T1 relaxation times were measured using the Bruker TopSpin pulse program t1ir (inversion-recovery) with an interscan delay (d1) of 10 s, an acquisition time (AQ) of 3.41 s, and 8 scans per increment. A total of 10 inversion delay intervals were used.

T2 relaxation times were measured using the Bruker TopSpin pulse program cpmg (Carr–Purcell–Meiboom–Gill) with an interscan delay (d1) of 10 s, an acquisition time (AQ) of 2.05 s, and 4 scans per increment. A total of 10 echo delay intervals were used.

Detailed fitting procedures and representative decay curves are provided in the T1 and T2 measurement reports.

T1 measurement reports of [N-$^{13}CH_3$]PtdCho and [N-$^{12}CH_3$]PtdCho

----

AREA fit :

I[t]=I[0]+P*exp(-t/T1)

10 points for Integral 1, Integral Region from 3.498 to 3.321 ppm; [N-$^{12}CH_3$]PtdCho

Results Comp. 1

I[0] = -9.988e-01

P = 1.917e+00

T1 = 458.168m

SD = 1.028e-03

tau	ppm	integral	intensity
10.000m	3.411	3.9596e+09	1.64e+08
50.000m	3.411	3.2476e+09	1.3617e+08
100.000m	3.411	2.4408e+09	1.0445e+08
200.000m	3.411	1.0763e+09	4.9157e+07
400.000m	3.411	-8.9737e+08	-3.1327e+07
800.000m	3.411	-2.9929e+09	-1.1669e+08
1.000s	3.411	-3.5243e+09	-1.3843e+08
2.000s	3.411	-4.3922e+09	-1.728e+08
3.000s	3.411	-4.4966e+09	-1.779e+08
5.000s	3.411	-4.5112e+09	-1.7877e+08

AREA fit :

I[t]=I[0]+P*exp(-t/T1)

10 points for Integral 1, Integral Region from 3.563 to 3.439 ppm; [N-$^{13}CH_3$I]PtdCho

Results Comp. 1

I[0] = -9.935e-01

P = 1.894e+00

T1 = 322.167m

SD = 6.689e-03

tau	ppm	integral	intensity
10.000m	3.494	1.5171e+10	2.0011e+08
50.000m	3.494	1.1174e+10	1.4897e+08
100.000m	3.494	6.9198e+09	9.4096e+07
200.000m	3.494	3.1296e+08	8.4412e+06

400.000m	3.494	-7.9456e+09	-9.8074e+07
800.000m	3.494	-1.4719e+10	-1.852e+08
1.000s	3.494	-1.603e+10	-2.0218e+08
2.000s	3.494	-1.7671e+10	-2.2291e+08
3.000s	3.494	-1.783e+10	-2.2468e+08
5.000s	3.494	-1.7835e+10	-2.2507e+08

---

10 points for Integral 2, Integral Region from 3.356 to 3.246 ppm; [N-$^{13}CH_3$r]PtdCho

Results Comp. 1

I[0] = -9.925e-01

P = 1.887e+00

T1 = 351.427m

SD = 9.135e-03

tau	ppm	integral	intensity
10.000m	3.313	1.5218e+10	2.0221e+08
50.000m	3.313	1.1449e+10	1.534e+08

100.000m	3.313	7.4319e+09	1.0134e+08
200.000m	3.314	1.1497e+09	1.9474e+07
400.000m	3.313	-6.8842e+09	-8.4806e+07
800.000m	3.313	-1.3981e+10	-1.7646e+08
1.000s	3.313	-1.5504e+10	-1.962e+08
2.000s	3.313	-1.761e+10	-2.2319e+08
3.000s	3.313	-1.7787e+10	-2.2577e+08
5.000s	3.313	-1.7805e+10	-2.263e+08

T2 measurement reports of [N-$^{13}CH_3$]PtdCho and [N-$^{12}CH_3$]PtdCho

----

AREA fit :

I[t]=P*exp(-t/T2)

10 points for Integral 1, Integral Region from 3.482 to 3.333 ppm; [N-$^{12}CH_3$]PtdCho

Results Comp. 1

P = 1.211e+000

T2 = 213.147m

SD = 2.240e-003

tau	ppm	integral	intensity
40.000m	3.410	7.9601e+009	1.617e+008
100.000m	3.410	6.0752e+009	1.215e+008
200.000m	3.410	3.7639e+009	7.6023e+007
300.000m	3.410	2.354e+009	4.7372e+007
400.000m	3.410	1.4659e+009	2.9556e+007
600.000m	3.410	5.7559e+008	1.1549e+007
800.000m	3.410	2.2364e+008	4.5248e+006
1.600s	3.410	7.0402e+006	1.2083e+005
2.000s	3.410	-9.4583e+005	eliminated
4.000s	3.409	-2.0906e+006	-27068

AREA fit :

I[t]=P*exp(-t/T2)

10 points for Integral 1, Integral Region from 3.567 to 3.432 ppm; [N-$^{13}CH_3$l]PtdCho

Results Comp. 1

P = 1.372e+00

T2 = 128.192m

SD = 2.926e-03

tau	ppm	integral	intensity
40.000m	3.495	3.2788e+10	3.086e+08
100.000m	3.495	2.0771e+10	1.9329e+08
200.000m	3.495	9.546e+09	8.7858e+07
300.000m	3.495	4.1828e+09	3.8822e+07
400.000m	3.495	1.8544e+09	1.717e+07
600.000m	3.496	3.8226e+08	3.3322e+06
800.000m	3.496	7.5228e+07	6.3677e+05
1.600s	3.495	4.3125e+06	eliminated
2.000s	3.495	5.0421e+06	eliminated

4.000s	3.495	55023	eliminated

AREA fit :

I[t]=P*exp(-t/T2)

10 points for Integral 2, Integral Region from 3.365 to 3.244 ppm; [N-$^{13}CH_3$r]PtdCho

Results Comp. 1

P = 1.357e+00

T2 = 134.290m

SD = 4.633e-03

tau	ppm	integral	intensity
40.000m	3.314	3.3304e+10	3.1389e+08
100.000m	3.314	2.1805e+10	1.9964e+08
200.000m	3.314	1.0204e+10	9.3646e+07
300.000m	3.314	4.684e+09	4.284e+07
400.000m	3.313	2.1282e+09	1.9611e+07

600.000m	3.313	4.5723e+08	4.1086e+06
800.000m	3.313	9.1014e+07	8.6787e+05
1.600s	3.314	2.7665e+06	eliminated
2.000s	3.314	-4.842e+06	eliminated
4.000s	3.314	2.7369e+06	eliminated

Necessity of band-selective INEPT for quantitative HMBC of [N-$^{13}CH_3$]PtdCho

As described in the main text, band-selective INEPT was employed to ensure complete polarization transfer from both N-$^{12}CH_3$ and N-$^{13}CH_3$ protons to ^{13}C1. Here, we provide a detailed theoretical analysis of the ^{13}C-^{13}C J-coupling effect and its suppression via the proposed pulse sequence design.

With a conventional HMBC pulse sequence, the density operator before t_2 acquisition differs fundamentally between the two molecular species. For natural abundance [N-$^{12}CH_3$]PtdCho, the coherence evolves as:

$$-\hat{I}_y\hat{E}_{C1} \rightarrow -2\hat{I}_x\hat{S}_{1z}\cos(\Omega_{C1}t_1)\sin(\pi J_{CH1}\tau)$$

Where $\hat{I}_y$ and $\hat{I}_x$ are the proton spin operators along the y- and x-axes, respectively, $\hat{S}_{1z}$ is the z-axis spin operator of ^{13}C1 (N-$^{13}CH_2$), $\hat{E}_{C1}$ is the identity operator of the ^{13}C1 nucleus, Ω_{C1} is the chemical shift of ^{13}C1 in angular frequency units, J_{CH1} is the three-bond ^{1}H-^{13}C J-coupling constant between the N-CH_3 proton and ^{13}C1, and τ is the INEPT delay optimized for ^{1}H-^{13}C1 polarization transfer ($\tau = 1/2J_{CH1}$).

For [N-$^{13}CH_3$]PtdCho, the N-$^{13}CH_3$ proton has three additional ^1H-^{13}C J-coupling interactions with the neighboring ^{13}C methyl nuclei, represented by identity operators $\hat{E}_a$, $\hat{E}_b$, $\hat{E}_c$, which introduce additional modulation terms:

$$-\hat{I}_z\hat{E}_a\hat{E}_b\hat{E}_c\hat{S}_{x1} \rightarrow -2\hat{I}_z\hat{S}_{1y}\cos(\Omega_{C1}t_1)\sin(\pi J_{CH1}\Delta)\prod_{k=a,b,c}\cos(\pi J_k\Delta)$$

where J_k (k = a, b, c) denotes the additional ^1H-^{13}C J-coupling constants between the N-$^{13}CH_3$ proton and the neighboring ^{13}C methyl nuclei: J_a is the one-bond coupling to the directly attached methyl ^{13}C, and J_b, J_c are the three-bond couplings to the remaining two methyl ^{13}C nuclei. The resulting HMBC signal ratio between [N-$^{13}CH_3$]PtdCho and [N-$^{12}CH_3$]PtdCho becomes:

$$\frac{x}{1-x}\cdot\prod_{k=a,b,c}\cos(\pi J_k\Delta)$$

where $x/(1-x)$ is the molar ratio of ^{13}C-labeled to natural abundance PtdCho. Since the product term is always less than unity due to the cosine modulation from each additional coupling, conventional HMBC systematically underestimates the [N-$^{13}CH_3$]PtdCho signal relative to [N-$^{12}CH_3$]PtdCho, rendering quantitative comparison between the two species fundamentally impossible without correction. By employing band-selective INEPT matched to the $^{13}C1$ resonance, all additional coupling evolutions are suppressed, reducing the product term to unity and restoring the true signal ratio of $x/(1-x)$ for both species.

^{13}C-^{13}C coupling effects and pulse sequence design for clean quantitative signals

Following the band-selective INEPT, the coherence resides on the $^{13}C1$ operator $-2\hat{I}_z\hat{S}_{1y}$, which represents an anti-phase state with respect to ^1H. Although this coherence is observable in t_2, it

gives rise to an anti-phase signal that causes severe signal cancellation due to overlap between components of opposite sign. A reversed INEPT sequence is therefore introduced to convert the anti-phase operator $-2\hat{I}_y\hat{C}_{1z}$ into an in-phase ^{1}H magnetization $\hat{I}_x$, simultaneously transferring the coherence back to the ^{1}H spin and ensuring a purely absorptive in-phase signal in t_2. However, both C1 and C2 carbons in PtdCho, produced from [U-^{13}C]ethanolamine and [U-^{13}C]choline as precursors, respectively, exist as ^{13}C isotopes regardless of the metabolic pathway, introducing ^{13}C1-^{13}C2 J-coupling that must be carefully managed across the pulse sequence.

In a conventional gradient-based HMBC experiment, a coherence selection gradient pair is incorporated within the t_1 encoding period to suppress undesired coherence pathways and reduce t_1-noise. To accommodate this gradient pair, an additional constant delay δ is necessarily introduced within the t_1 evolution period. As both C1 and C2 carbons in PtdCho, produced from [U-^{13}C]ethanolamine and [U-^{13}C]choline as precursors, respectively, exist as ^{13}C isotopes regardless of the metabolic pathway, this additional delay δ allows further evolution of the ^{13}C1-^{13}C2 J-coupling beyond the t_1 encoding period, introducing phase distortion that must be carefully suppressed

Without the selective refocusing pulse on ^{13}C1, the full signal form of the ^{13}C1-correlated HMBC is:

$$S(t_1,t_2) \propto \cos(\Omega_{C1}t_1)\cos(\pi J_{12}(t_1+\delta)) \cdot \sin^2(\pi J_{CH1}\Delta)$$
$$\cdot \exp(i\Omega_H t_2)\cos(\pi J_{CH1}t_2) \prod_{k=a,b,c} \cos(\pi J_k t_2)$$

where Ω_H is the chemical shift of the N-CH_3 proton in angular frequency units, t_2 is the F2 direct acquisition time, J_{12} is the $^{13}C1$-$^{13}C2$ one-bond J-coupling constant, and δ is the coherence selection gradient delay. The term $\cos(\pi J_{12}(t_1 + \delta))$ can be expanded as:

$$\cos(\pi J_{12}(t_1 + \delta)) = \cos(\pi J_{12} t_1)\cos(\pi J_{12}\delta) - \sin(\pi J_{12} t_1)\sin(\pi J_{12}\delta)$$

This coexistence of cosine- and sine-modulated J_{12} terms in t_1 gives rise to a mixture of absorptive and dispersive lineshapes in the F1 dimension, resulting in phase-distorted signals that prevent accurate quantitative analysis. To suppress this phase distortion while preserving the t_1-encoded J_{12} evolution, a selective refocusing pulse on $^{13}C1$ (N-$^{13}CH_2$) was introduced during the gradient delay period δ, refocusing the J_{12} coupling evolution over δ and restoring the pure cosine-modulated form. Furthermore, any residual anti-phase magnetization remaining after the reversed INEPT sequence is converted into unobservable multiple-quantum (MQ) coherence by the final ^{13}C pulse — the "Clean" element of the CLIP (Clean In-Phase) INEPT sequence — ensuring that only a purely absorptive in-phase signal contributes to the detected HMBC response.

As a result of all the above corrections, the final signal forms for the three molecular species are:

Endogenous (natural abundance) PtdCho:

$$S^{endo}(t_1, t_2) \propto \cos(\Omega_{C1} t_1) \cdot \sin^2(\pi J_{CH1}\delta) \cdot \exp(i\Omega_H t_2)\cos(\pi J_{CH1} t_2)$$

[N-$^{12}CH_3$]PtdCho (from [U-^{13}C]ethanolamine):

$$S^{12C}(t_1, t_2) \propto \cos(\Omega_{C1} t_1)\cos(\pi J_{12} t_1) \cdot \sin^2(\pi J_{CH1}\delta) \cdot \exp(i\Omega_H t_2)\cos(\pi J_{CH1} t_2)$$

[N-^{13}CH$_3$]PtdCho (from [U-^{13}C]choline):

$$S^{13C}(t_1,t_2) \propto \cos(\Omega_{C1} t_1)\cos(\pi J_{12} t_1) \cdot \sin^2(\pi J_{HC1} \tau)$$

$$\cdot \exp(i\Omega_H t_2)\cos(\pi J_{HC1} t_2) \prod_{k=a,b,c} \cos(\pi J_k t_2)$$

These three signal forms enable a two-level spectral discrimination. First, endogenous unlabeled PtdCho, which lacks the $\cos(\pi J_{12} t_1)$ term, appears as a singlet in the F1 dimension, whereas both ^{13}C-tracer-derived species appear as doublets at $(\Omega_{C1} \pm J_{12}/2)$, enabling clear separation from the endogenous background. Second, among the two labeled species, [N-^{12}CH$_3$]PtdCho and [N-^{13}CH$_3$]PtdCho are distinguished by their F2 signal multiplicity. It should be noted that J_{HC1}(<8 Hz), being a three-bond ^{1}H-^{13}C coupling constant, is too small to be resolved as a splitting in the F2 dimension under typical 2D NMR resolution conditions and effectively appears as a singlet for both species. In contrast, the one-bond ^{1}H-^{13}C coupling J_k (~145 Hz) of the N-^{13}CH$_3$ group dominates the F2 multiplet pattern of [N-^{13}CH$_3$]PtdCho, giving rise to a clearly resolved doublet. Consequently, [N-^{12}CH$_3$]PtdCho and [N-^{13}CH$_3$]PtdCho are practically distinguished as a singlet versus doublet in the F2 dimension.

Product-operator description of the modified HMBC sequence

Spin system and definitions

I = N-CH$_3$ proton; S_1 = ^{13}C1 (N-^{13}CH$_2$); S_2 = ^{13}C2 (O-^{13}CH$_2$); $C_{a,b,c}$ = the three methyl ^{13}C nuclei.

J_{HC1} = ^{3}J(I–S_1) drives the transfer; J_{12} = ^{1}J(S_1–S_2) ≈ 40 Hz; $^1J_{CH}$ ≈ 145 Hz (one-bond I-C$_{methyl}$ coupling); In this derivation we take $\tau = 1/(4\ ^3J_{HC1})$ for ideal polarization transfer; experimentally

τ = 21.44 ms was set by the SNR/√T optimization (shorter than $1/(4\ ^3J_{HC1})$, $^3J_{HC1} \approx 7$ Hz, to limit T_2 losses)$\tau = 1/(4\ J_{HC1})$.

Derivation — [N-^{12}CH$_3$] (PEMT) and [N-^{13}CH3] (Kennedy) compared

Both species have ^{13}C-labelled C1 and C2, so J_{12} and the J_{HC1} transfer are common to both; they differ only in the ^{12}C / ^{13}C status of the N-methyl. Spin operators are written with a hat; in the linear lines below, they appear as $\hat{□}$ and $\hat{□}$. Equilibrium: $\rho_0 = \hat{□}_□$ (the ^{13}C polarization is negligible).

Common steps (identical for both species):

(1) $1.08 \times 90°_□$ (^{1}H): $\hat{□}_□ \rightarrow -\hat{□}_□$

(2) band-sel. INEPT, 2τ: $2\hat{□}_□\hat{□}_{1□}$ (selective 180° on ^{13}C1 only)

(3) $90°_{□1_□}$ (^{13}C): $-2\ \hat{□}_□\hat{□}_{1□}$

(4) Δ (sel. 180° on ^{13}C1): $2\hat{□}_□\hat{□}_{1□}$ *^{13}C1 shift, $□(\hat{□} - \hat{□}_1)$ and $□_{12}$ refocused; gradient encodes

(5) $□_1$ (^{1}H 180° center)

: $2\hat{□}_□[\{\hat{□}_{1□}\cos(□_1\ □_1) - \hat{□}_{1□}\sin(□_1\ □_1)\}\cos(□\ □_{12}\ □_1) + \{-2\hat{□}_{1□}\hat{□}_{2□}\cos(□_1\ □_1) - 2\hat{□}_{1□}\hat{□}_{2□}\sin(□_1\ □_1)\}\sin(□\ □_{12}\ □_1)]$

(6) Δ (sel. 180° on ^{13}C1)

:$-2\,\hat{□}_{□}[\{\hat{□}_{1□}\cos(□_1\,□_1)+\hat{□}_{1□}\sin(□_1\,□_1)\}\cos(□\,□_{12}\,□_1)+\{-2\hat{□}_{1□}\hat{□}_{2□}\cos(□_1\,□_1)+$ $2\hat{□}_{1□}\hat{□}_{2□}\sin(□_1\,□_1)\}\sin(□\,□_{12}\,□_1)]$ * $□_{12}$ is refocused over Δ; gradient decodes

(7) $90°_{□2_□}$(^{13}C)

: $-2\hat{□}_{□}\hat{□}_{1□}\cos(□_1□_1)\cos(□□_{12}□_1)$

* Only single quantum (SQ) AP can be observed in the acquisition after the rest of pulse sequence.

(8) band-sel. reversed INEPT + CLIP

: $\hat{□}_{□}\cos(□_1\,□_1)\cos(□□_{12}□_1)+$ * residual Multiple quantum (MQ) state (purged)

Detection (decoupler off) — where the two species diverge:

[N-^{12}CH3] (PEMT): $\propto\cos(□_1□_1)\cos(□□_{12}□_1)\exp(□□_{□}□_2)$

[N-^{13}CH3] (Kennedy): $\propto\cos(□_1□_1)\cos(□□_{12}□_1)\exp(□□_{□}□_2)\cos(□\cdot{}^1□_{□□}□_2)$

For completeness, the pre-existing (natural-abundance) species lacks ^{13}C at C2, so the J_{12} factor is absent and it is a singlet in both dimensions:

Pre-existing: $\propto\cos(□_1□_1)\exp(□□_{□}□_2)$

Quantitative correction of ^{13}C-isotope-dependent relaxation effects in HMBC spectroscopy

Quantitative correction of ^{13}C-isotope-dependent relaxation effects in HMBC spectroscopy

Conventional two-dimensional ^1H–^{13}C correlation NMR experiments require repeated measurements to achieve sufficient sensitivity and F1 resolution, with an interscan delay (d1) allowing recovery of nuclear spin populations via T1 relaxation. In contrast to NMR-inactive ^{12}C, ^{13}C adjacent to a ^1H spin introduces additional intramolecular dipole–dipole interactions with the N–CH_3 protons, constituting a major relaxation pathway. Consequently, the T1 and T2 relaxation constants of N-$^{13}CH_3$ protons differ from those of N-$^{12}CH_3$, leading to systematic signal distortion in HMBC. When d1 is shorter than the full relaxation condition (d1 $\geq$ 5T1), longitudinal magnetization imbalance accumulates over successive scans, resulting in systematic errors in relative signal intensities. In typical experiments on small molecules such as PtdCho, d1 is often set to less than 2 s to maximize sensitivity per unit time, necessitating post-acquisition correction.

T1 and T2 relaxation constant measurements

To quantitatively model these relaxation effects, the T1 and T2 relaxation constants of the N–CH_3 protons in [N-$^{12}CH_3$]PtdCho and [N-$^{13}CH_3$]PtdCho were experimentally determined using their respective standard compounds dissolved in $CDCl_3$, on the same 800 MHz spectrometer used for all experiments (see Materials section). The measured values are summarized in Table S1.

Table S1. T1 and T2 relaxation constants for [N-$^{12}CH_3$]PtdCho and [N-$^{13}CH_3$]PtdCho

Species	T1 (s)	T2 (s)
[N-$^{12}CH_3$]PtdCho	0.458	0.213
[N-$^{13}CH_3$r]PtdCho	0.351	0.134

[N-$^{13}CH_3$l]PtdCho	0.322	0.129

Here, [N-$^{13}CH_3$r]PtdCho and [N-$^{13}CH_3$l]PtdCho denote the right and left doublet peaks observed in the F2 dimension of the ^{13}C1-correlated HMBC signal, respectively.

Bloch equation–based simulation framework

All numerical analyses were performed using a Bloch equation–based simulation framework that explicitly models the evolution of magnetization throughout the HMBC pulse sequence. The sequence was partitioned into discrete time segments defined by the 180° refocusing pulses, and magnetization was propagated through each segment by solving the Bloch equations.

T1 relaxation was incorporated in all segments, including both the pulse sequence elements and the inter-transient delay (acquisition + d1), to describe the continuous recovery of longitudinal magnetization toward thermal equilibrium.

In contrast, T2 relaxation was applied only to segments in which transverse magnetization is present, specifically during the coherence transfer periods prior to detection (e.g., INEPT delays). Signal intensity was calculated by propagating the magnetization through all segments and accumulating the transverse magnetization contributions over the full acquisition scheme (NS × TD transients).

Numerical optimization of acquisition parameters

Using this simulation framework, numerical optimization of SNR/√T was performed to maximize NMR signal sensitivity per unit time under the given HMBC acquisition conditions (NS and TD). The excitation angle, INEPT delay, and inter-transient delay (d1 + acquisition time) were treated as variables.

Average T1 and T2 values of the N-$^{13}CH_3$ protons were used during optimization. The procedure was iteratively performed using a simulated annealing algorithm [1]. Final acquisition parameters were selected considering both the numerical results and practical experimental constraints, as further improvements in SNR efficiency were marginal.

Correction factor calculation

Using the same simulation framework and the optimized acquisition parameters, correction factors were calculated by incorporating species-specific T1 and T2 relaxation constants. Simulations were performed independently for [N-$^{12}CH_3$]PtdCho, [N-$^{13}CH_3$r]PtdCho, and [N-$^{13}CH_3$l]PtdCho, and signal contributions were accumulated over all 3,072 transients.

The resulting cumulative signals were compared to derive correction factors. The simulation yielded correction factors of 1.097 and 1.086 for [N-$^{13}CH_3$r]PtdCho and [N-$^{13}CH_3$l]PtdCho, respectively (mean = 1.092), corresponding to an approximately 9% systematic underestimation of the ^{13}C-bearing species under the applied experimental conditions. These correction factors were applied to experimentally measured HMBC signal intensities prior to quantitative pathway analysis. Implementation details and the corresponding Python code are provided in this Supporting Information.

Python code for the correction factor calculation

```
"""
==============================================================================
hmbc_13C_signal_correction_factors.py
==============================================================================
Purpose:
    Compute signal correction factors for quantitative HMBC spectroscopy
    to account for ¹³C-induced relaxation imbalance between [N-¹²CH₃]PtdCho
    and [N-¹³CH₃]PtdCho (r and l isotopomers).

Method:
    Bloch equation simulation incorporating both T1 (spin-lattice) and
    T2 (spin-spin) relaxation over the full HMBC acquisition scheme
    (NS × TD transients). Correction factors are derived as the ratio of
    cumulative transverse magnetization of each ¹³C-bearing species
    relative to the ¹²C reference.

Output:
```

```
    - Cumulative signal for [N-¹²CH₃], [N-¹³CH₃r], and [N-¹³CH₃l] PtdCho

    - Correction factors for [N-¹³CH₃r] and [N-¹³CH₃l] relative to [N-¹²CH₃]


  Usage:

    python hmbc_13C_signal_correction_factors.py


  Author : [Jin Wook Cha]
═══════════════════════════════════════════════════════════════════════
"""


  import numpy as np


  #──Parameter setup ──────────────────────────────────────────────

  J = 11.6  # Hz

  J1 = 11.6

  JNCH = 7.0

  J  = float(input("Insert J: "))

  J1 = float(input("Insert J1: "))
```

```
DELTA  = 0.5 * (1 / (4 * J))   # INEPT delay (s)

DELTA1  = 0.5 * (1 / (4 * J1))  # INEPT delay for J1 (s)

base_pulse_4_73  = (1.19 + 2.33 + 1.18) / 1000  # Base pulse duration: 4.73 ms → seconds

post_pulse  = float(input("Insert post_time: "))  # Acq.+ d1 delay (s)

M_eq = 1.0  # Equilibrium magnetization (Mz at thermal equilibrium)


#──Relaxation constant inputs ──────────────────────────────

T1r = float(input("Enter T1r value: "))   # T1 for [N-13CH3r]PtdCho

T1l = float(input("Enter T1l value: "))   # T1 for [N-13CH3l]PtdCho

T1  = float(input("Enter T1 value: "))    # T1 for [N-12CH3]PtdCho

T2r = float(input("Enter T2r value: "))   # T2 for [N-13CH3r]PtdCho

T2l = float(input("Enter T2l value: "))   # T2 for [N-13CH3l]PtdCho

T2  = float(input("Enter T2 value: "))    # T2 for [N-12CH3]PtdCho

NS  = int(input("Enter NS value: "))      # Number of scans per increment

TD  = int(input("Enter TD1 value: "))     # Number of F1 increments


"""
```

```
# Reference values (for verification):

T1r = 0.350

T1l = 0.321

T1  = 0.458

T2r = 0.134

T2l = 0.129

T2  = 0.213

"""


repeats = NS * TD  # Total number of transients


#——Functions ——————————————————————————————

def calculate_t_k(k):

    """

    Calculate the t_1 evolution time t_k for the k-th transient.

    The evolution time increments every 2*NS transients.

    """
```

```
    return base_pulse_4_73 + (1.053 * 0.5 * (k // (NS * 2))) / 1000  # ms → s


def T1_relaxation(Mz_initial, T1, time):
    """
    Apply T1 (spin-lattice) relaxation to longitudinal magnetization Mz.
    Mz(t) = M_eq + (Mz_initial - M_eq) * exp(-t / T1)
    """
    return M_eq + (Mz_initial - M_eq) * np.exp(-time / T1)


def T2_relaxation(Mx_initial, T2, time):
    """
    Apply T2 (spin-spin) relaxation to transverse magnetization Mx.
    Mx(t) = Mx_initial * exp(-t / T2)
    """
    return Mx_initial * np.exp(-time / T2)


#──Bloch simulation for [N-12CH3]PtdCho ──────────────────────────
```

```
Mz_values_T1 = [M_eq]  # Longitudinal magnetization history
Mx_values_T1 = []      # Transverse (observable) magnetization per transient


for k in range(repeats):
    t_k = calculate_t_k(k)


    # Excitation pulse (flip angle ~1.08 × π/2)
    Mz_T1 = Mz_values_T1[k] * np.cos(1.08 * np.pi / 2)


    # Transverse magnetization after INEPT transfer and T2 relaxation over full evolution period
    Mx_T1 = (np.sin(4 * np.pi * JNCH * DELTA) * np.sin(4 * np.pi * JNCH * DELTA1)) * \
            T2_relaxation(Mz_values_T1[k] * np.sin(1.08 * np.pi / 2),
                          T2, 2 * t_k + 2 * DELTA + 2 * DELTA1)
    Mx_values_T1.append(Mx_T1)


    # T1 relaxation through the pulse sequence delays
    Mz_T1 = T1_relaxation(Mz_T1, T1, DELTA)
```

```
    Mz_T1 = -Mz_T1                          # 180° refocusing pulse

    Mz_T1 = T1_relaxation(Mz_T1, T1, DELTA)

    Mz_T1 = T1_relaxation(Mz_T1, T1, t_k)

    Mz_T1 = -Mz_T1                          # 180° refocusing pulse

    Mz_T1 = T1_relaxation(Mz_T1, T1, DELTA1)

    Mz_T1 = T1_relaxation(Mz_T1, T1, t_k)

    Mz_T1 = -Mz_T1                          # 180° refocusing pulse

    Mz_T1 = T1_relaxation(Mz_T1, T1, DELTA1 + post_pulse)   # Recovery during post-
acquisition delay

    Mz_values_T1.append(Mz_T1)


  #——Bloch simulation for [N-13CH3r]PtdCho ————————————————————————

  Mz_values_T1r = [M_eq]

  Mx_values_T1r = []


  for m in range(repeats):

    t_k = calculate_t_k(m)
```

```
Mz_T1 = Mz_values_T1r[m] * np.cos(1.08 * np.pi / 2)


Mx_T1 = (np.sin(4 * np.pi * JNCH * DELTA) * np.sin(4 * np.pi * JNCH * DELTA1)) * \
    T2_relaxation(Mz_values_T1r[m] * np.sin(1.08 * np.pi / 2),
          T2r, 2 * t_k + 2 * DELTA + 2 * DELTA1)
Mx_values_T1r.append(Mx_T1)


Mz_T1 = T1_relaxation(Mz_T1, T1r, DELTA)
Mz_T1 = -Mz_T1
Mz_T1 = T1_relaxation(Mz_T1, T1r, DELTA)
Mz_T1 = T1_relaxation(Mz_T1, T1r, t_k)
Mz_T1 = -Mz_T1
Mz_T1 = T1_relaxation(Mz_T1, T1r, DELTA1)
Mz_T1 = T1_relaxation(Mz_T1, T1r, t_k)
Mz_T1 = -Mz_T1
Mz_T1 = T1_relaxation(Mz_T1, T1r, DELTA1 + post_pulse)
Mz_values_T1r.append(Mz_T1)
```

```
#——Bloch simulation for [N-13CH3l]PtdCho ————————————————————————————————

Mz_values_T1l = [M_eq]

Mx_values_T1l = []


for j in range(repeats):

    t_k = calculate_t_k(j)


    Mz_T1 = Mz_values_T1l[j] * np.cos(1.08 * np.pi / 2)


    Mx_T1 = (np.sin(4 * np.pi * JNCH * DELTA) * np.sin(4 * np.pi * JNCH * DELTA1)) * \

            T2_relaxation(Mz_values_T1l[j] * np.sin(1.08 * np.pi / 2),

                          T2l, 2 * t_k + 2 * DELTA + 2 * DELTA1)

    Mx_values_T1l.append(Mx_T1)


    Mz_T1 = T1_relaxation(Mz_T1, T1l, DELTA)

    Mz_T1 = -Mz_T1
```

```
    Mz_T1 = T1_relaxation(Mz_T1, T1l, DELTA)
    Mz_T1 = T1_relaxation(Mz_T1, T1l, t_k)
    Mz_T1 = -Mz_T1
    Mz_T1 = T1_relaxation(Mz_T1, T1l, DELTA1)
    Mz_T1 = T1_relaxation(Mz_T1, T1l, t_k)
    Mz_T1 = -Mz_T1
    Mz_T1 = T1_relaxation(Mz_T1, T1l, DELTA1 + post_pulse)
    Mz_values_T1l.append(Mz_T1)

#──Results: cumulative signal and correction factors ──────────────────
sum_T1  = np.sum(Mx_values_T1)   # Total accumulated signal for [N-12CH3]PtdCho
sum_T1r = np.sum(Mx_values_T1r)  # Total accumulated signal for [N-13CH3r]PtdCho
sum_T1l = np.sum(Mx_values_T1l)  # Total accumulated signal for [N-13CH3l]PtdCho


print(f'N-12CH3:  {sum_T1}')
print(f'N-13CH3r: {sum_T1r}')
print(f'N-13CH3l: {sum_T1l}')
```

```
# Correction factors relative to [N-12CH3]PtdCho

print(f'Correction factor for N-13CH3l: {sum_T1l / sum_T1}')

print(f'Correction factor for N-12CH3: {sum_T1 / sum_T1}')   # Reference (= 1.000)

print(f'Correction factor for N-13CH3r: {sum_T1r / sum_T1}')
```

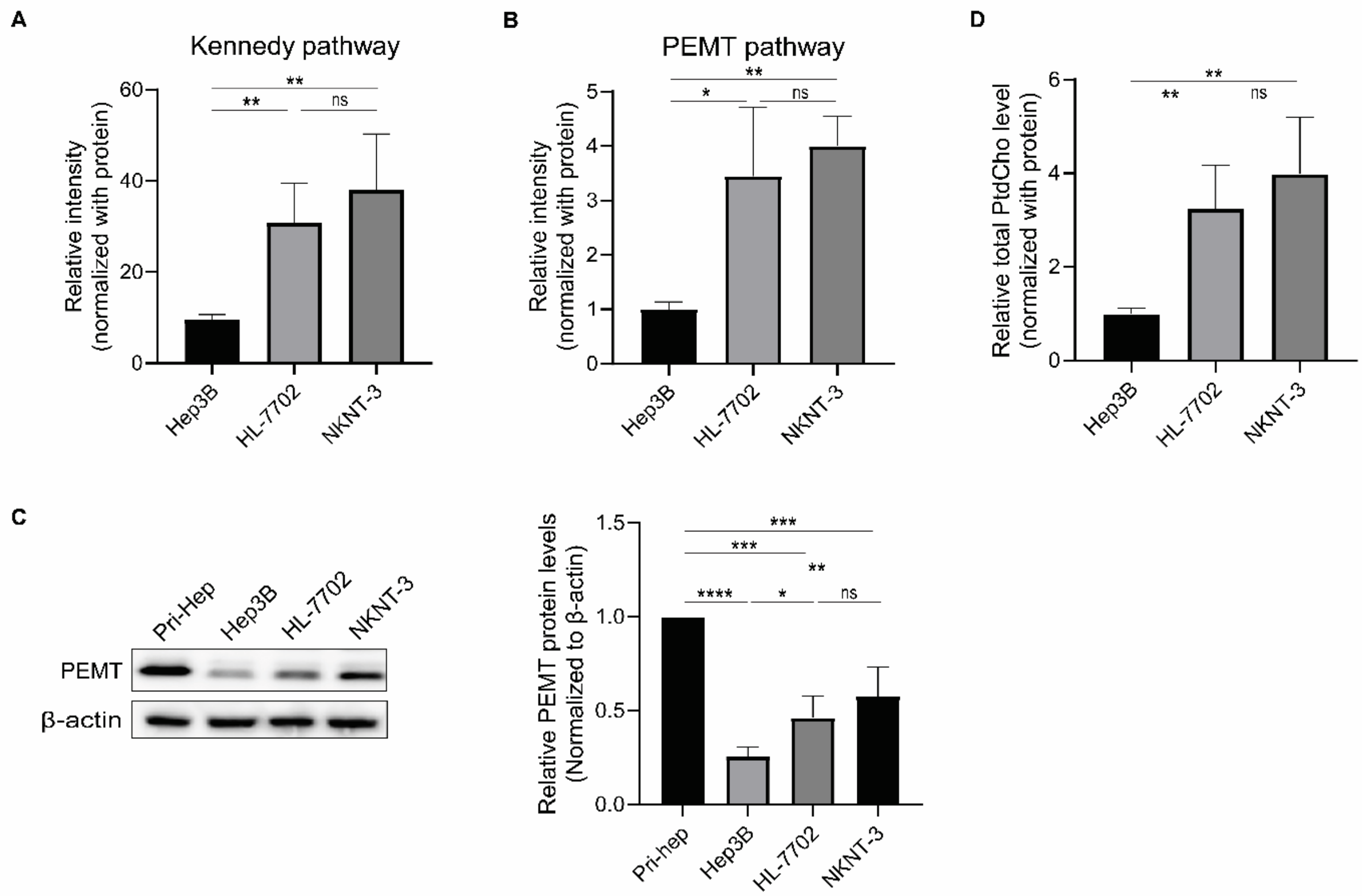

Figure S1. Kennedy pathway activity and PEMT pathway activity in various liver cells

A. Relative intensity of PtdCho synthesized from the Kennedy pathway in liver cells. Data were analyzed using ordinary one-way ANOVA, and a post hoc two-stage step-up method of Benjamini, Krieger, and Yekutieli was applied for multiple comparisons.

B. Relative intensity of PtdCho synthesized from the PEMT pathway in liver cells. Data were analyzed using ordinary one-way ANOVA, and a post hoc two-stage step-up method of Benjamini, Krieger, and Yekutieli was applied for multiple comparisons.

C. Western blot analysis of PEMT level in liver cells, normalized by β-actin.

D. Relative intensity of total PtdCho level in liver cells. Data were analyzed using ordinary one-way ANOVA, and a post hoc two-stage step-up method of Benjamini, Krieger, and Yekutieli was applied for multiple comparisons.

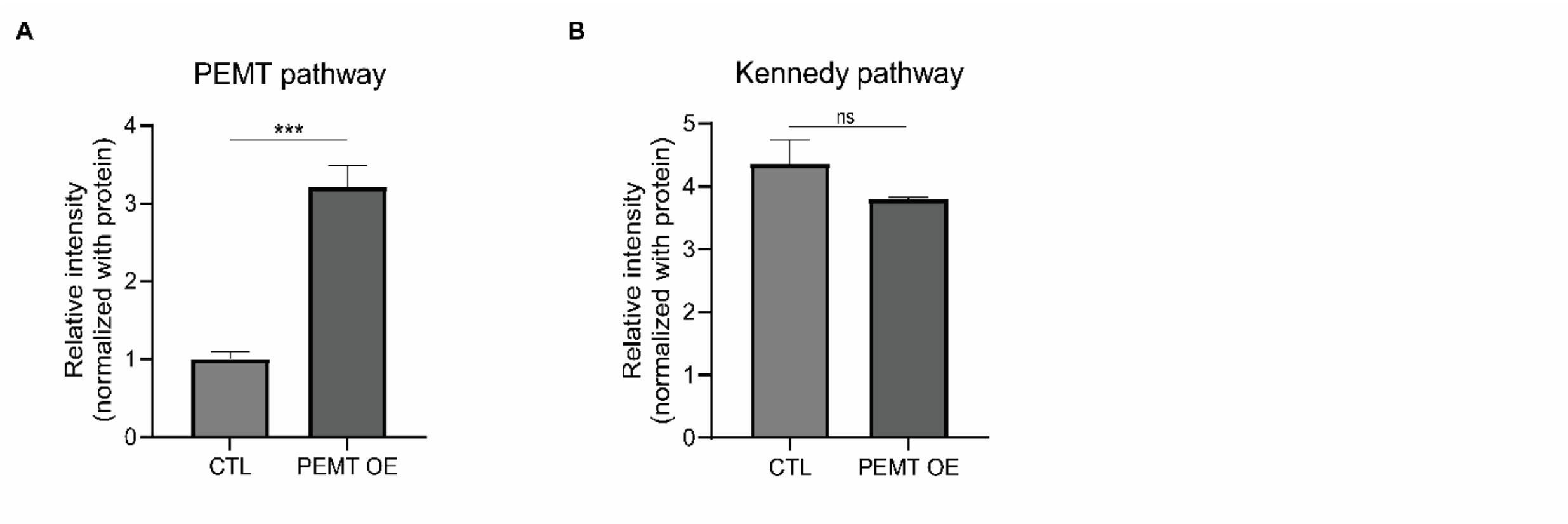

Figure S2. Kennedy pathway activity and PEMT pathway activity in CTL and PEMT OE cell

A. Relative intensity of PtdCho synthesized from the PEMT pathway in Hep3B CTL and PEMT OE cells. Data were analyzed by un-paired *t*-test.

B. Relative intensity of PtdCho synthesized from the Kennedy pathway in Hep3B CTL and PEMT OE cells. Data were analyzed by un-paired *t*-test.

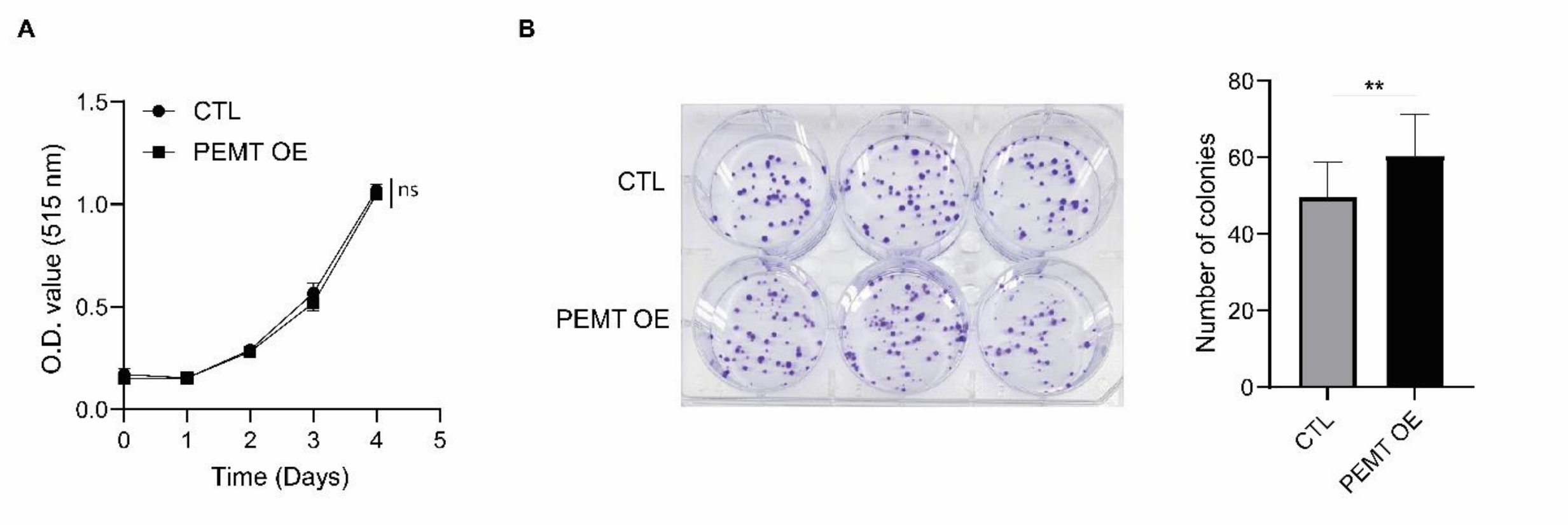

Figure S3. Proliferation of CTL and PEMT OE Hep3B cells

A. Proliferation of Hep3B CTL and PEMT OE cells measured with SRB assay. Data were analyzed by un-paired *t*-test.

B. Colony formation assay of Hep3B CTL and PEMT OE cells and its quantification graph was analyzed by un-paired *t*-test.

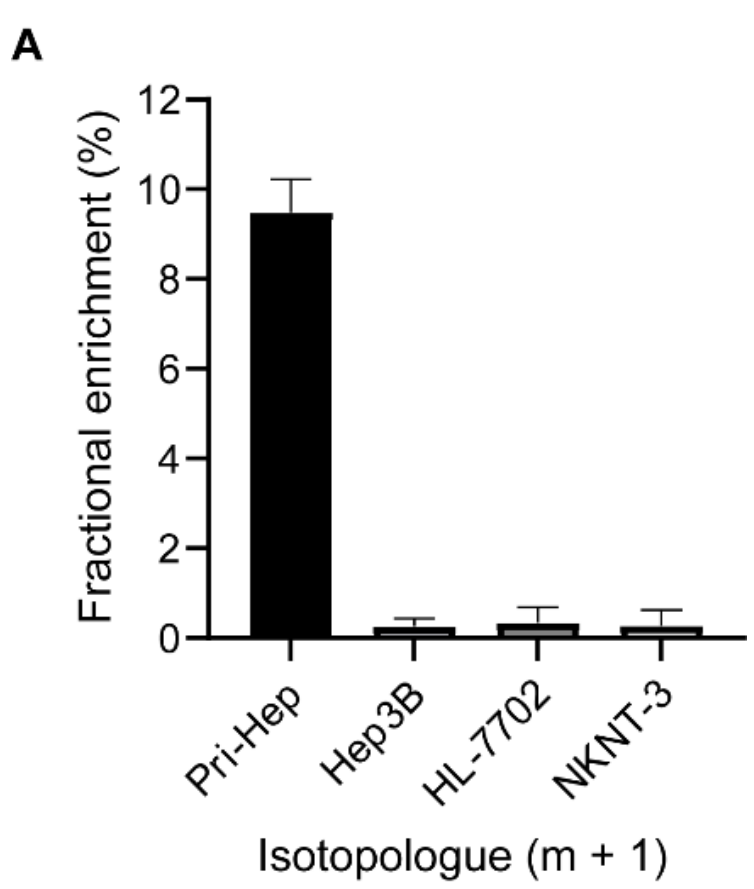

Figure S4. Effect of choline-PEMT contribution to total PEMT-generated PtdCho

A. Fractional enrichment of SAM isotopologue (m + 1) in various liver cells measured by LC-MS. Fractional enrichment of the isotopologue (m + 1) excludes a contribution from the natural ^{13}C abundance.

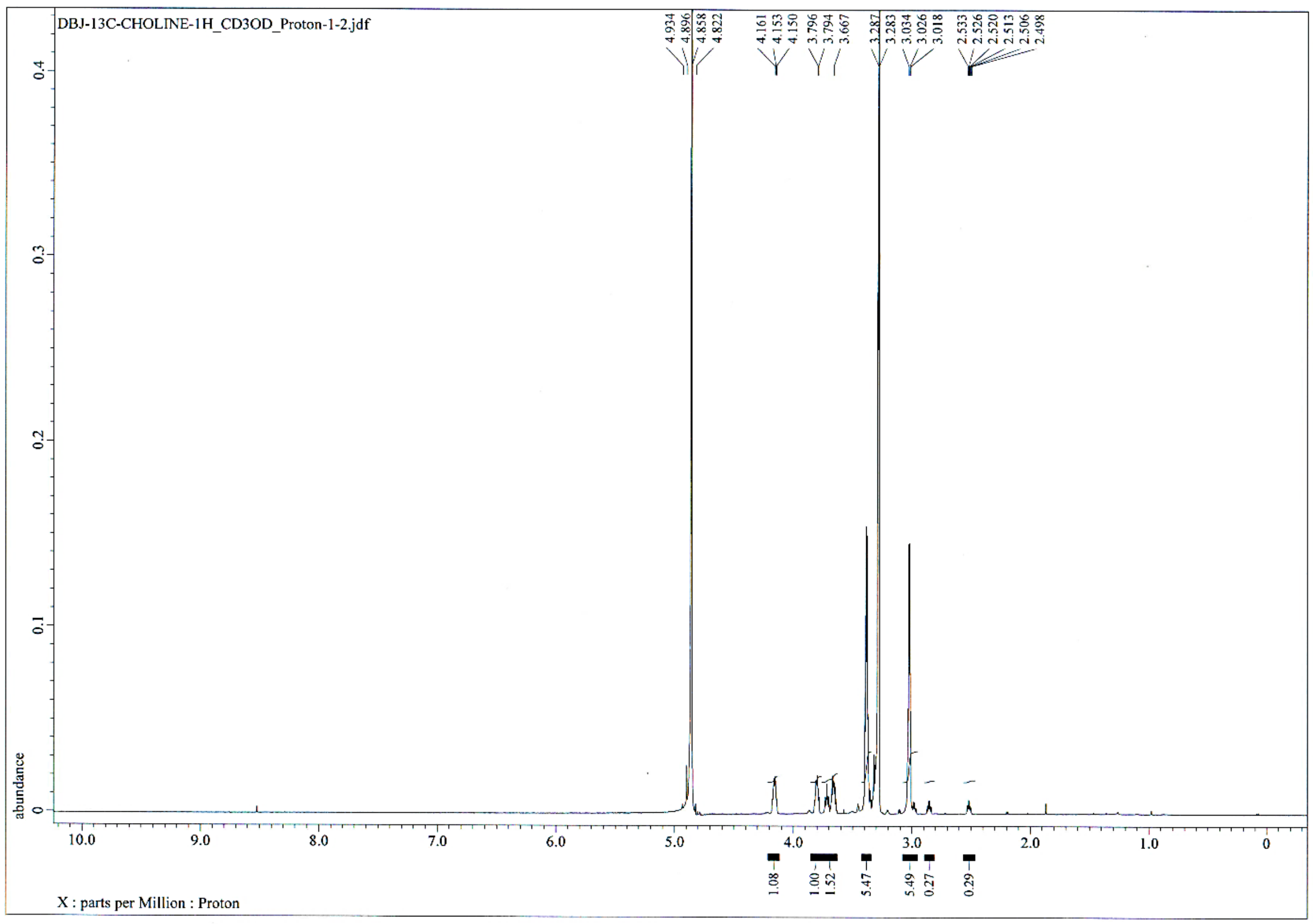

Figure S5: The ^{1}H NMR spectrum of **2** in CD_3OD (400 MHz)

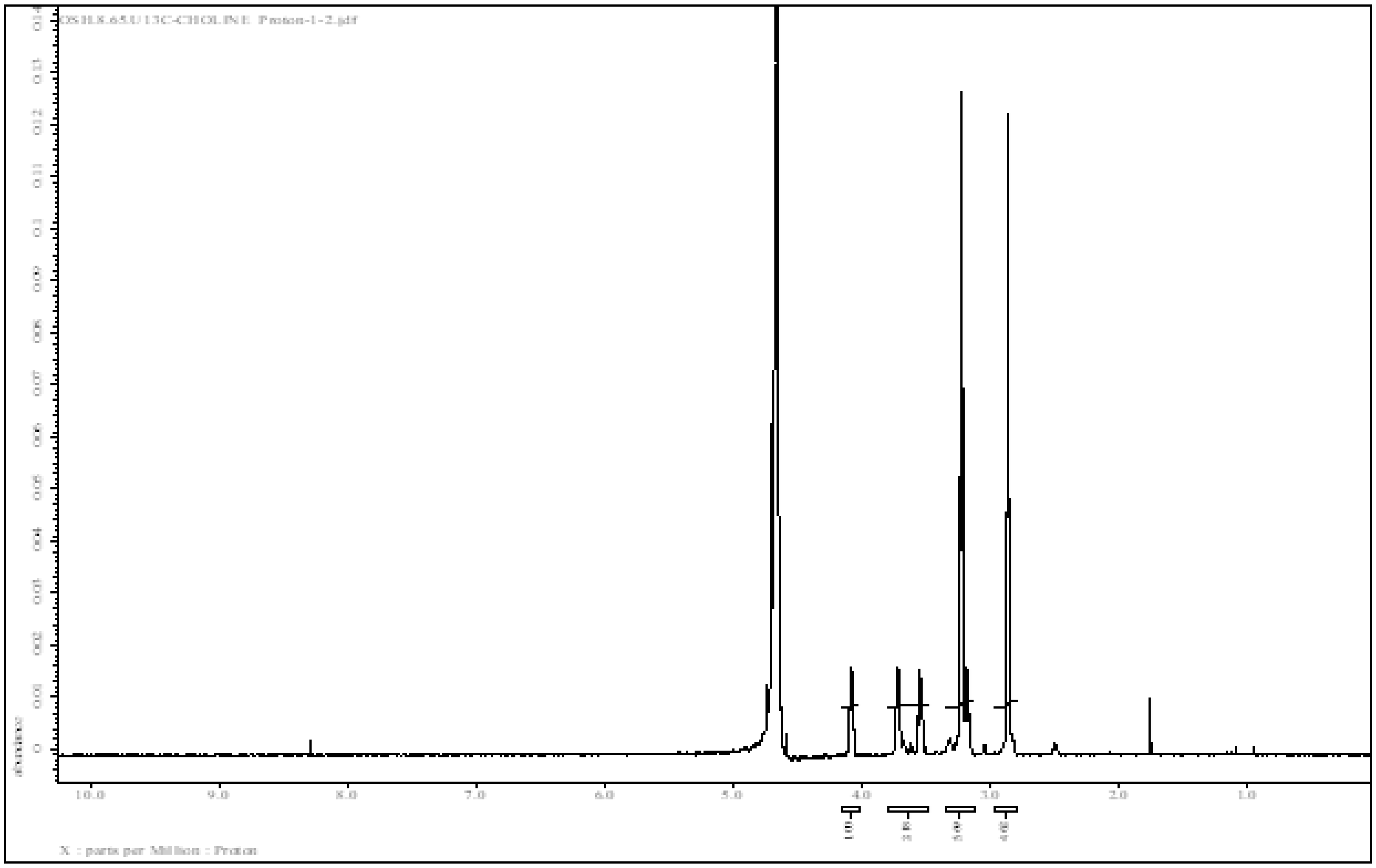

Figure S6: The ^{1}H NMR spectrum of **2** in D_2O (400 MHz)

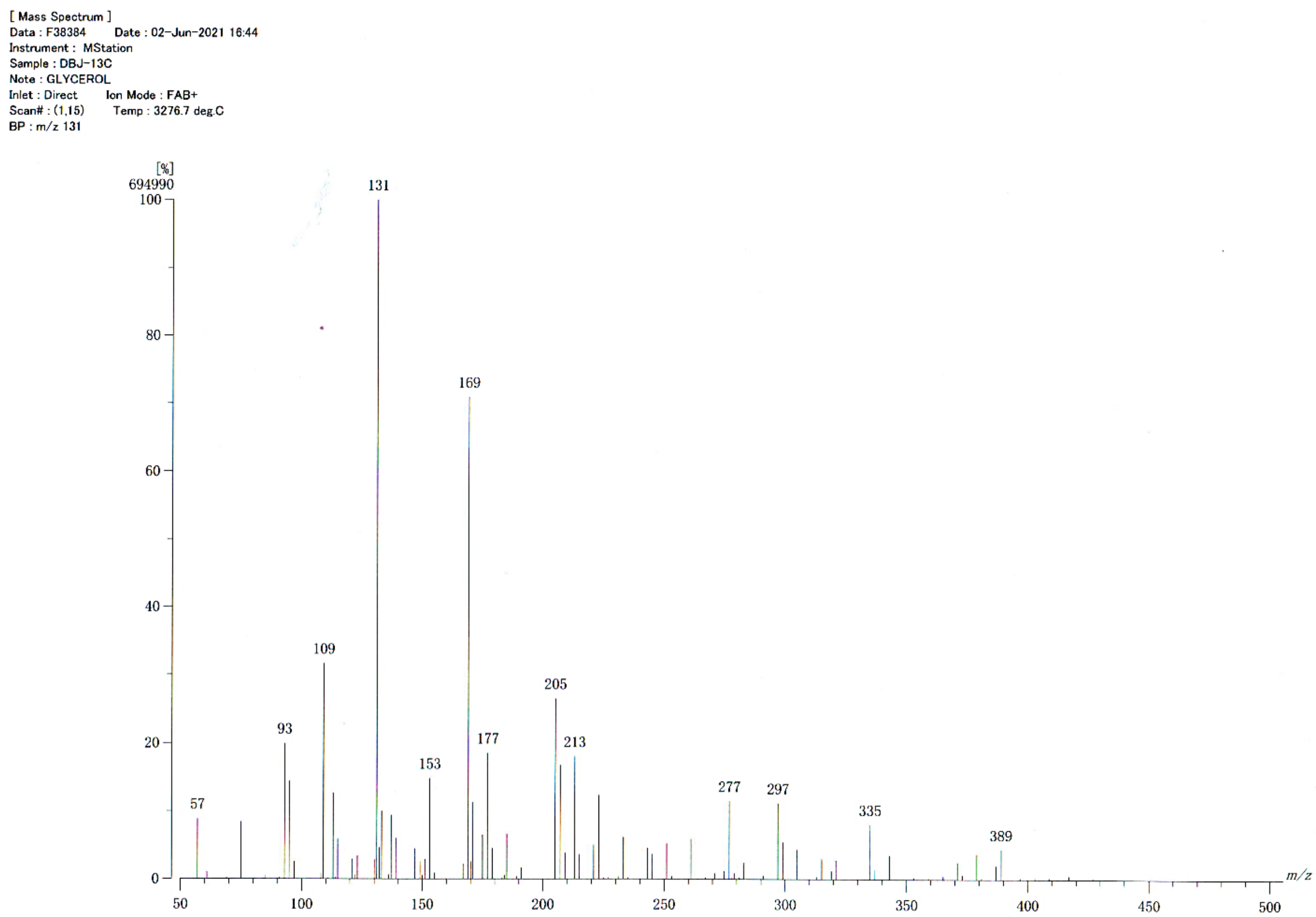

Figure S7: The mass spectrum of **2**

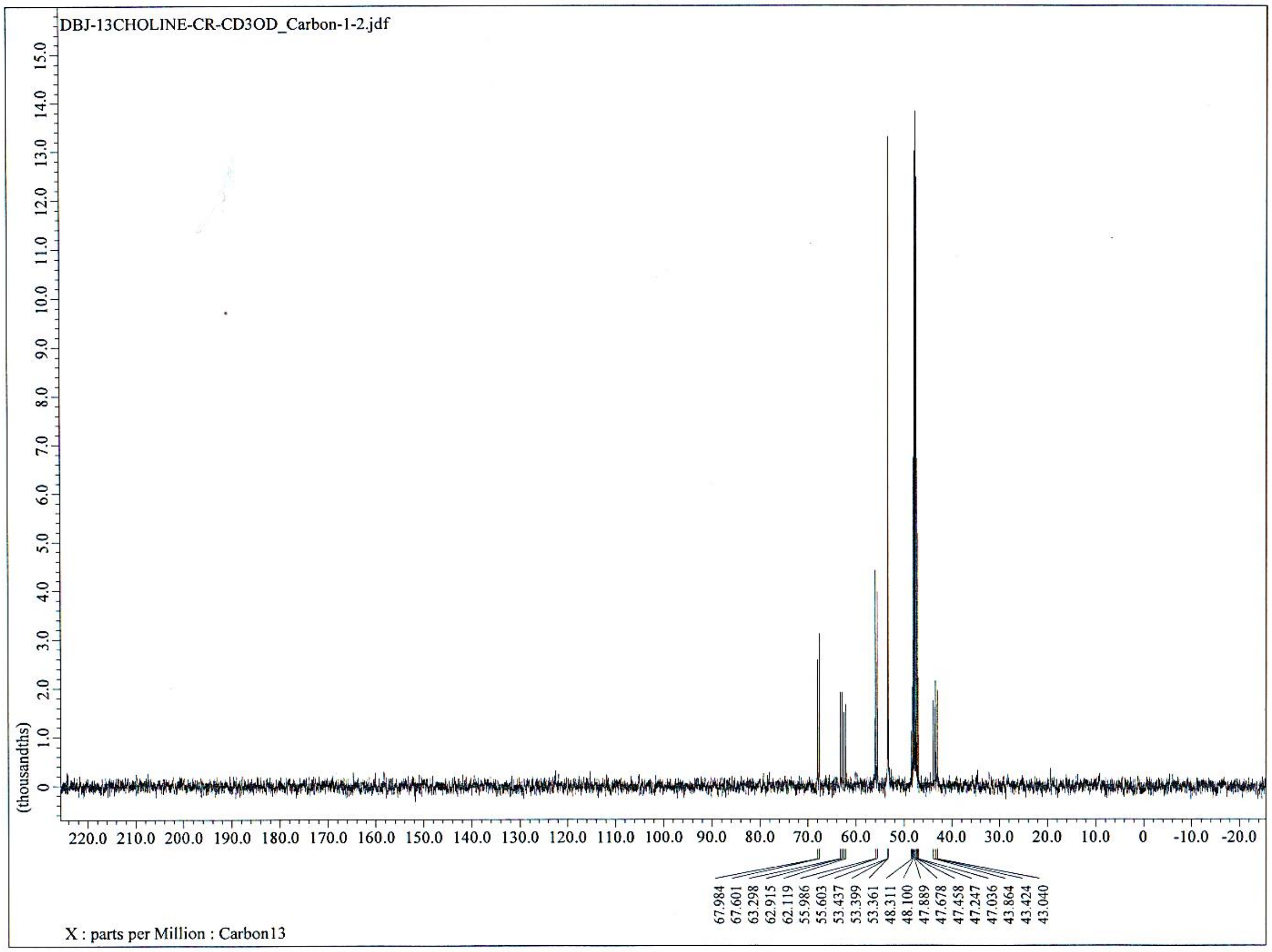

Figure S8: The ^{13}C NMR spectrum of **2** in CD_3OD (100 MHz)

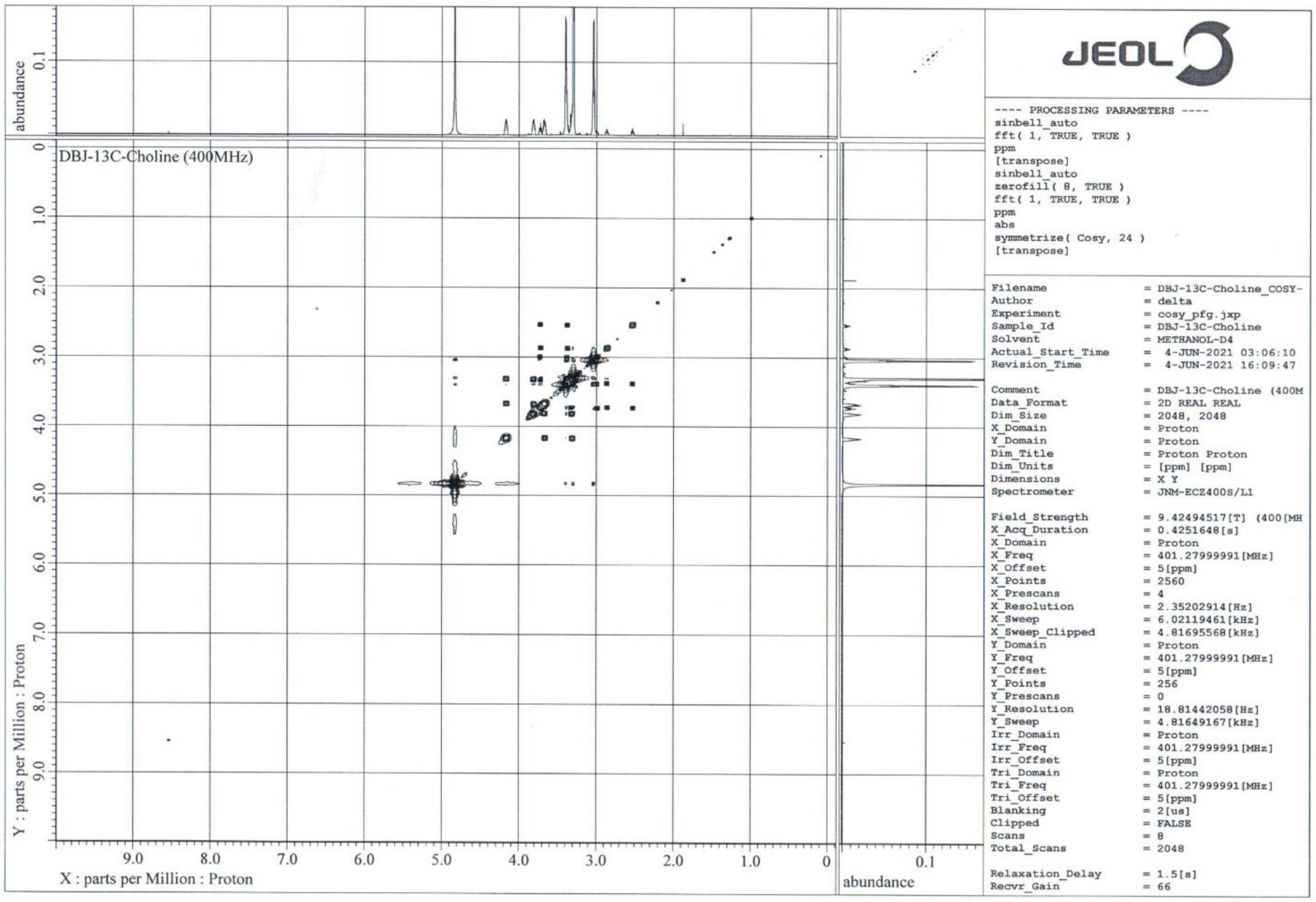

Figure S9: The ^{1}H-^{1}H COSY NMR spectrum of **2** (400 MHz)

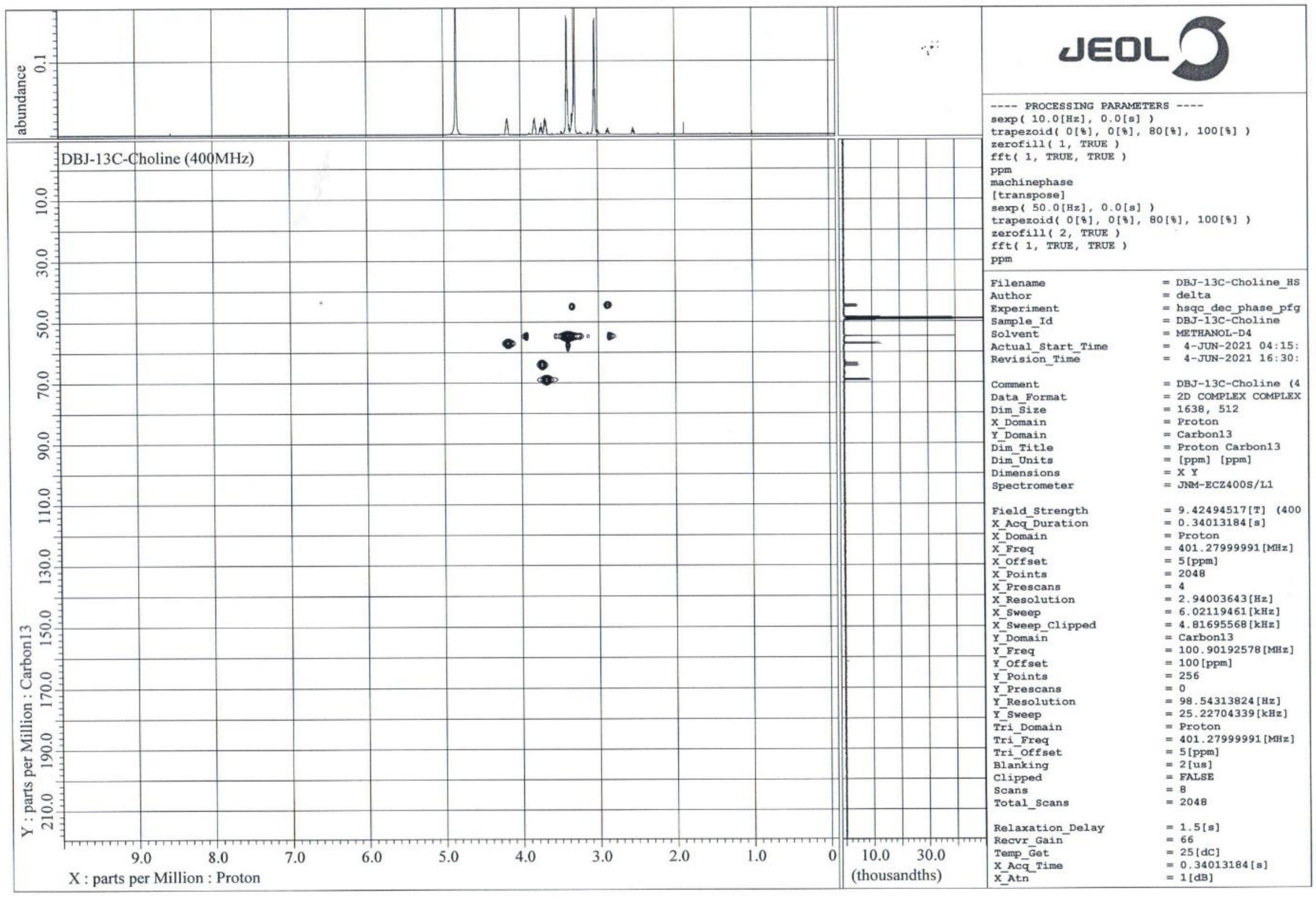

Figure S10: The ^{1}H-^{13}C HSQC NMR spectrum of **2** (400 MHz)

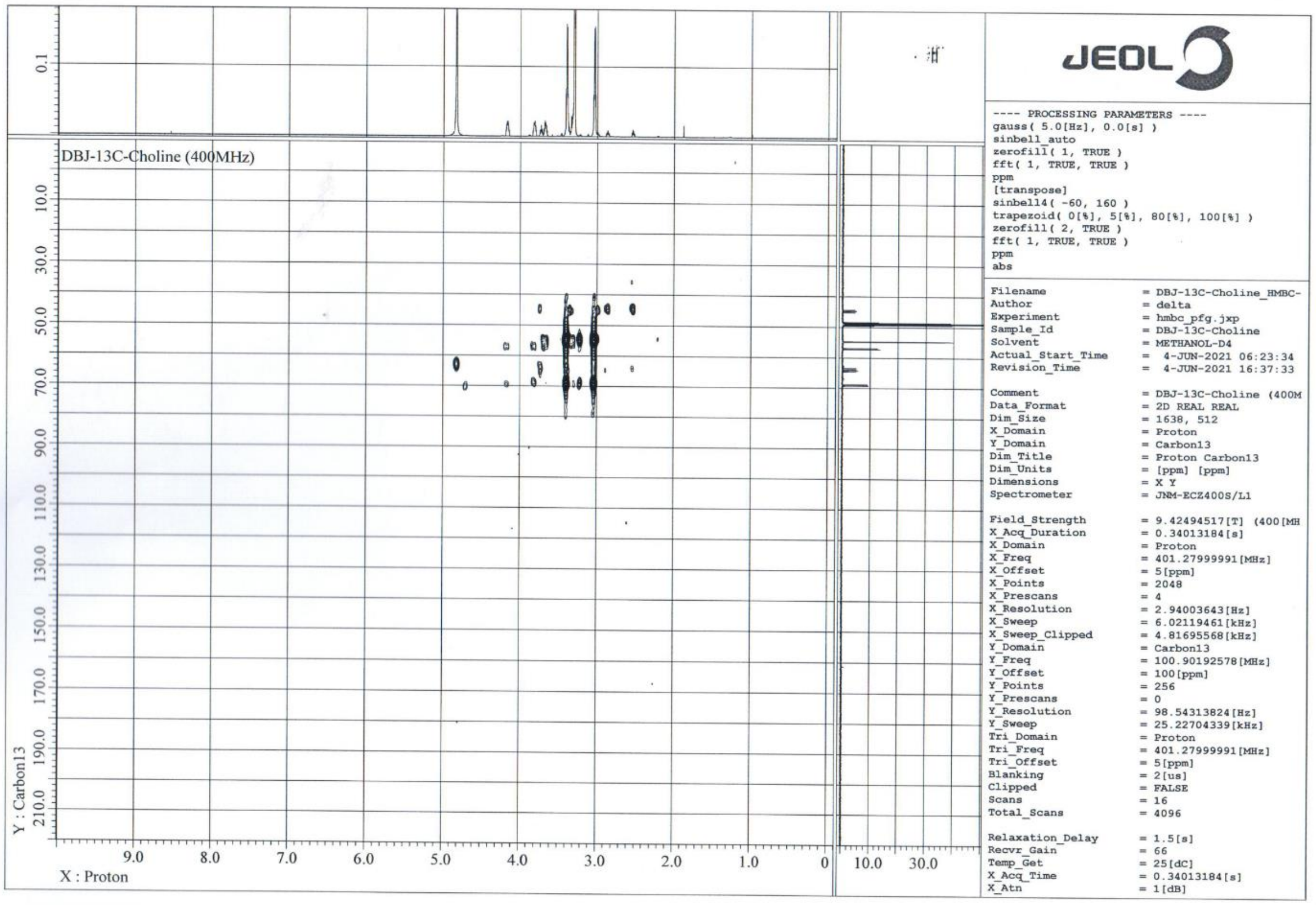

Figure S11: The ^{1}H-^{13}C HMBC NMR spectrum of **2** (400 MHz)

Table S2: NMR assignment of **2**

Position	δ_C (ppm), mult	δ_H (ppm), mult (J)	^{1}H satellites (δ, ppm)	COSY	HMBC (H→C)
C1, HO**CH**$_2$	55.8, m (55.99, 55.60)	3.97, m ($^1J_{CH}$ ≈ 145 Hz)	4.17–4.14 & 3.82–3.78	H1↔H2	H1→C2
C2, **CH**$_2$N$^+$	67.8, m (67.98, 67.60)	3.50, m	3.75–3.65 & 3.32–3.30	H2↔H1	H2→C1
N$^+$(**CH**$_3$)$_3$	53.4, m (53.44, 53.36)	3.21, m ($^1J_{CH}$ ≈ 145 Hz, ^2J = 3.2 Hz)	3.41–3.38 & 3.08–3.00	–	(CH$_3$)→C2

HSQC ($^1J_{CH}$ one-bond): C1(55.8)–H1(3.97), C2(67.8)–H2(3.50), N$^+$(CH$_3$)$_3$(53.4)–H(3.21).

Impurities-residual starting material: trace U-^{13}C ethanolamine **(1)** (minor signals at δ_C ~63 and ~43; δ_H approx. ~3.7 and ~3.0) does not affect the choline assignment.

Conditions: CD_3OD, (^{1}H 400, ^{13}C 100 MHz); shifts are uncorrected peak-pick values, consistent with the figures. With ~99% ^{13}C labeling all signals are multiplets: in ^{1}H the central ^{12}C line is absent and the ^{13}C satellites ($^1J_{CH}$ ≈ 145 Hz) are observed, so δ_H values are satellite-pair centers; in ^{13}C each carbon is split by $^1J_{CC}$ ≈ 38 Hz (N-methyl shows none, C–N$^+$–C). OH not observed; residual solvent δ_H ~3.3, δ_C ~47.7.

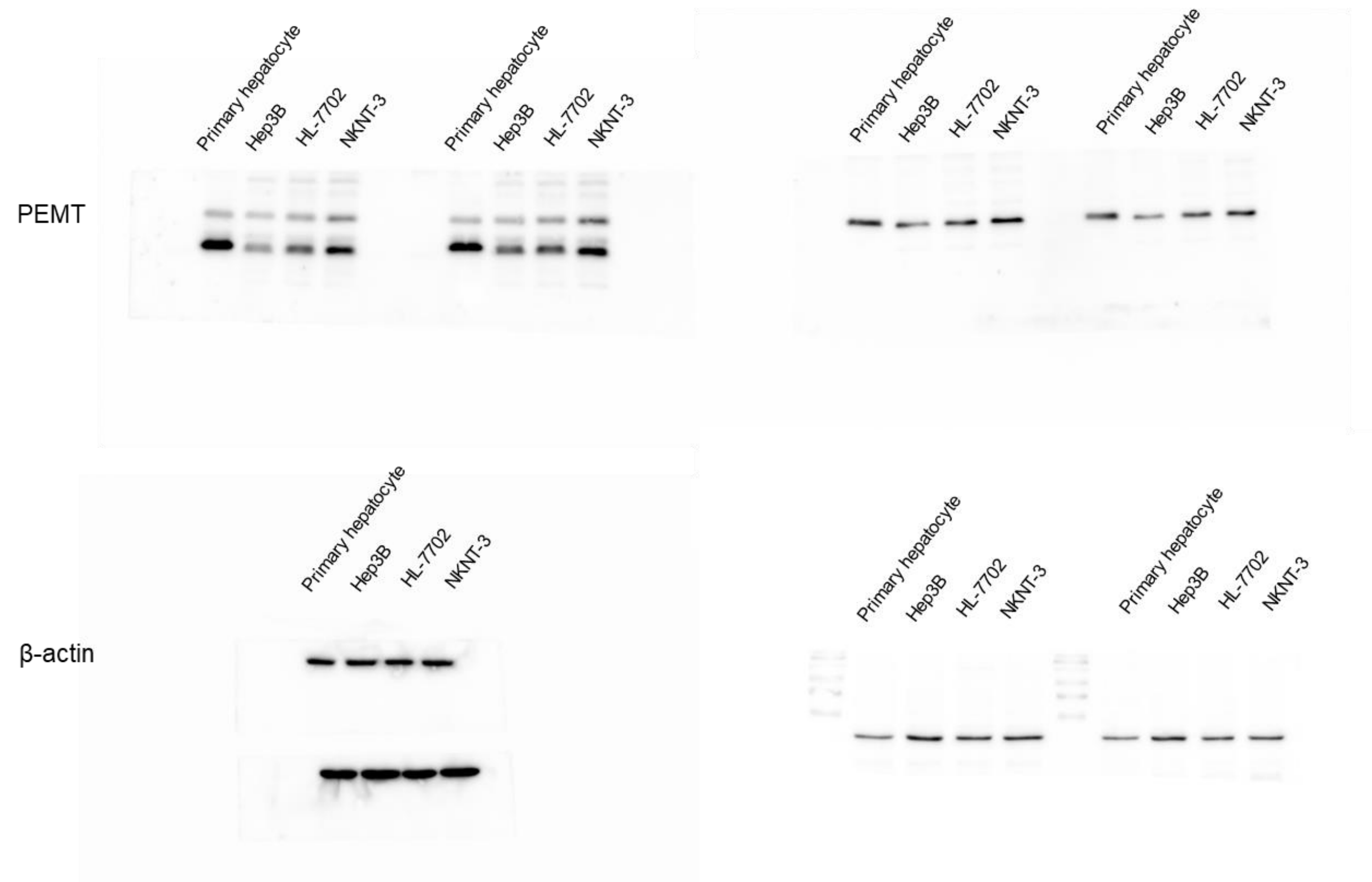

Figure S12: Original western blots for Figure S1C

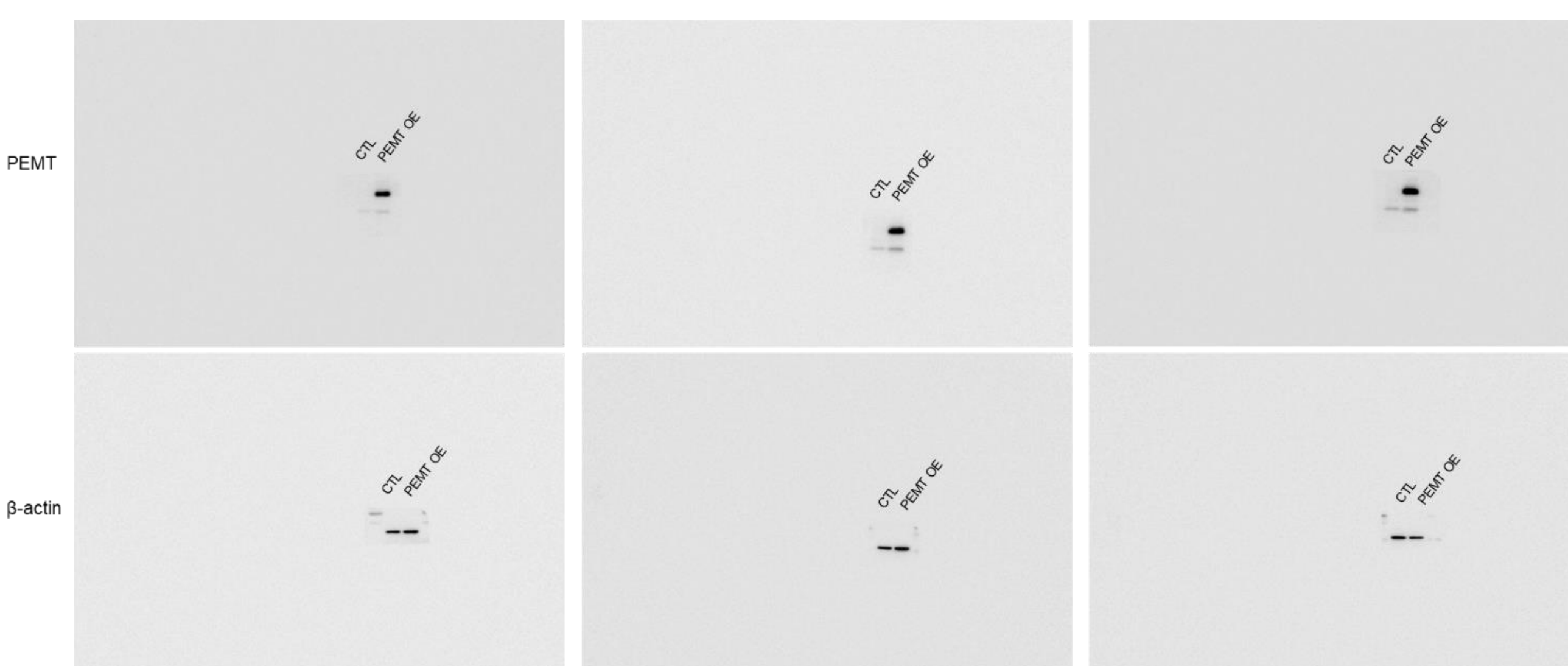

Figure S13: Original western blots for Figure 4A

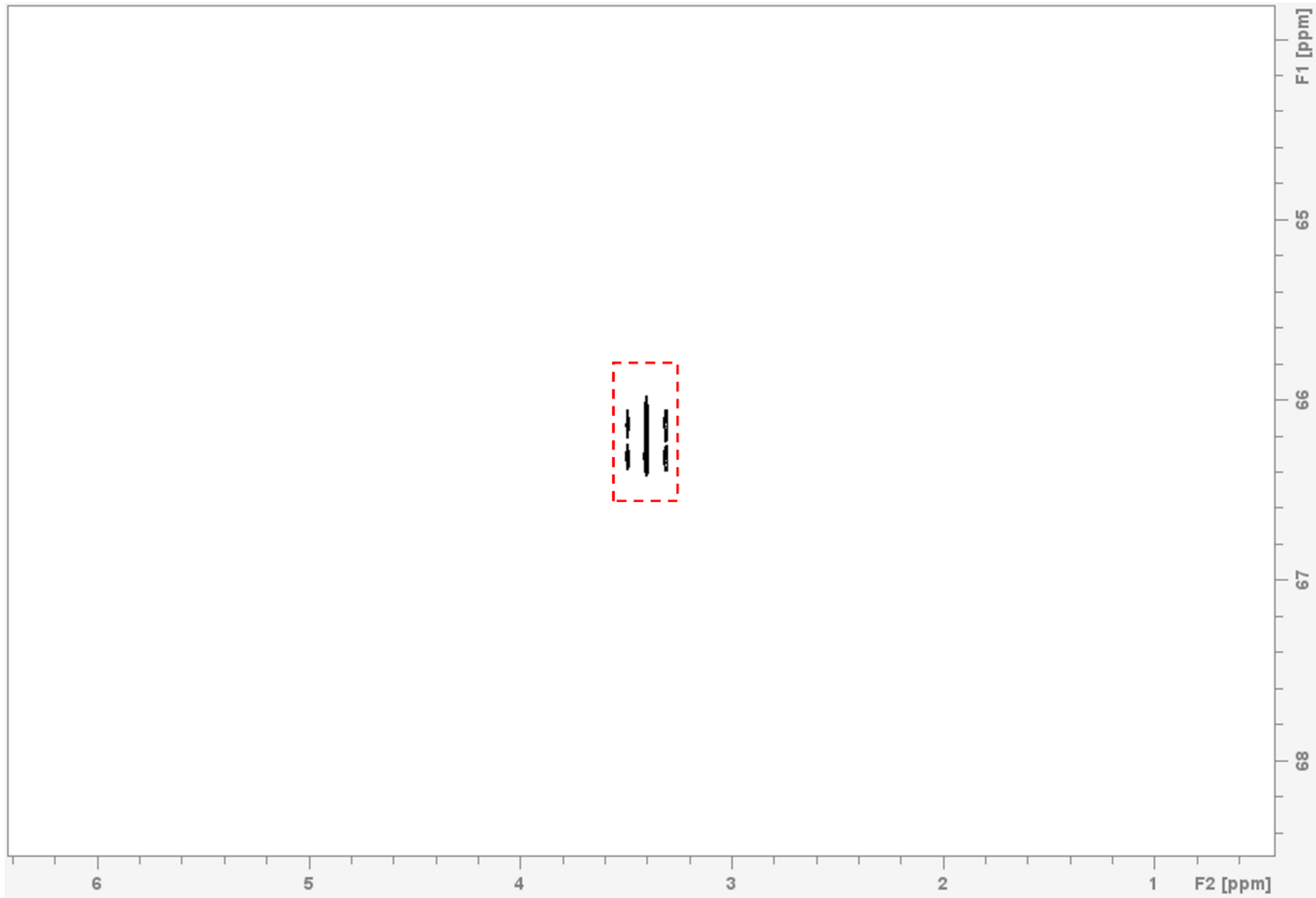

Figure S14: Full 2D HMBC spectrum corresponding to Figure 3A (Pri-Hep). The dashed box indicates the region of interest.

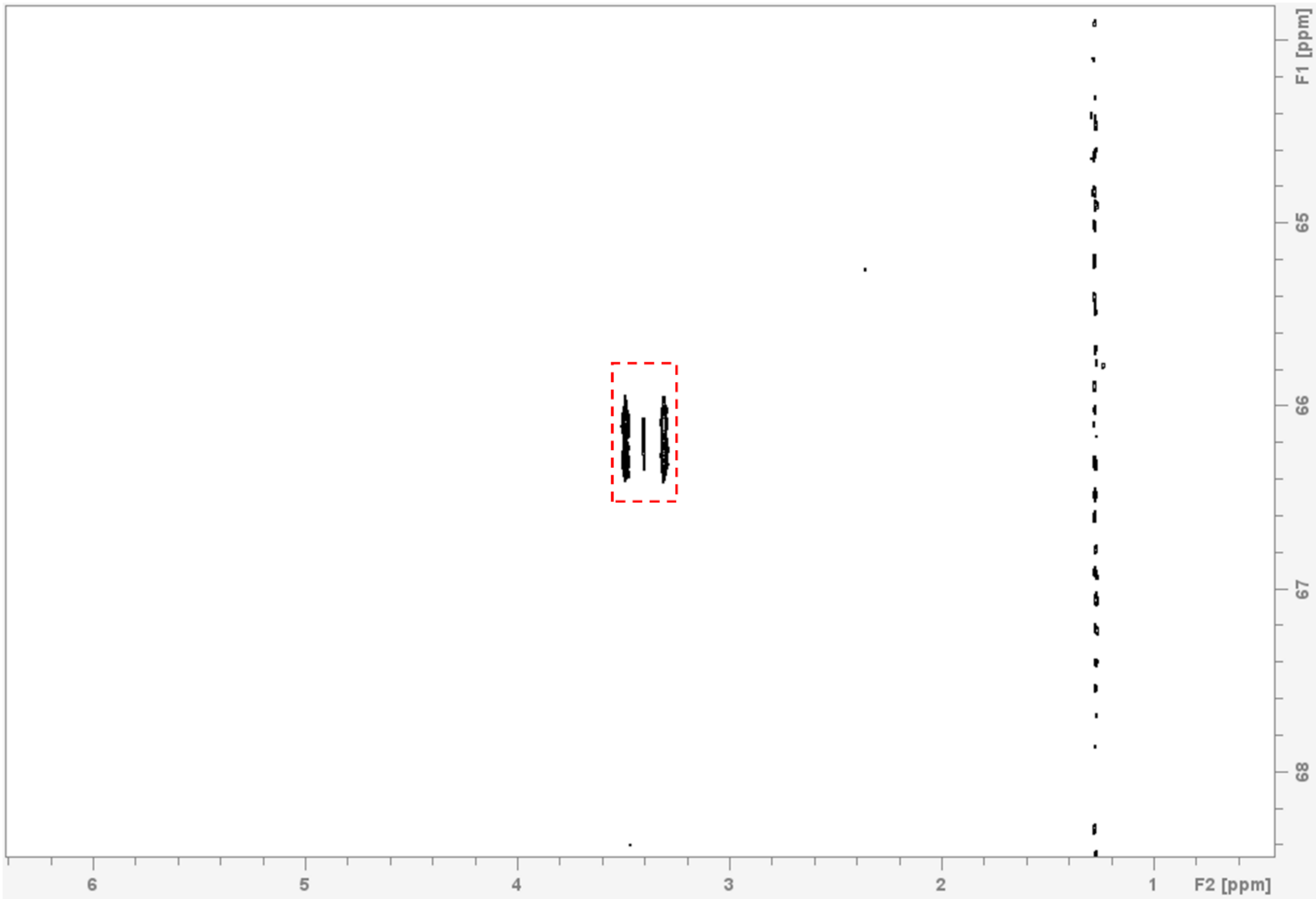

Figure S15: Full 2D HMBC spectrum corresponding to Figure 3A (HL-7702). The dashed box indicates the region of interest.

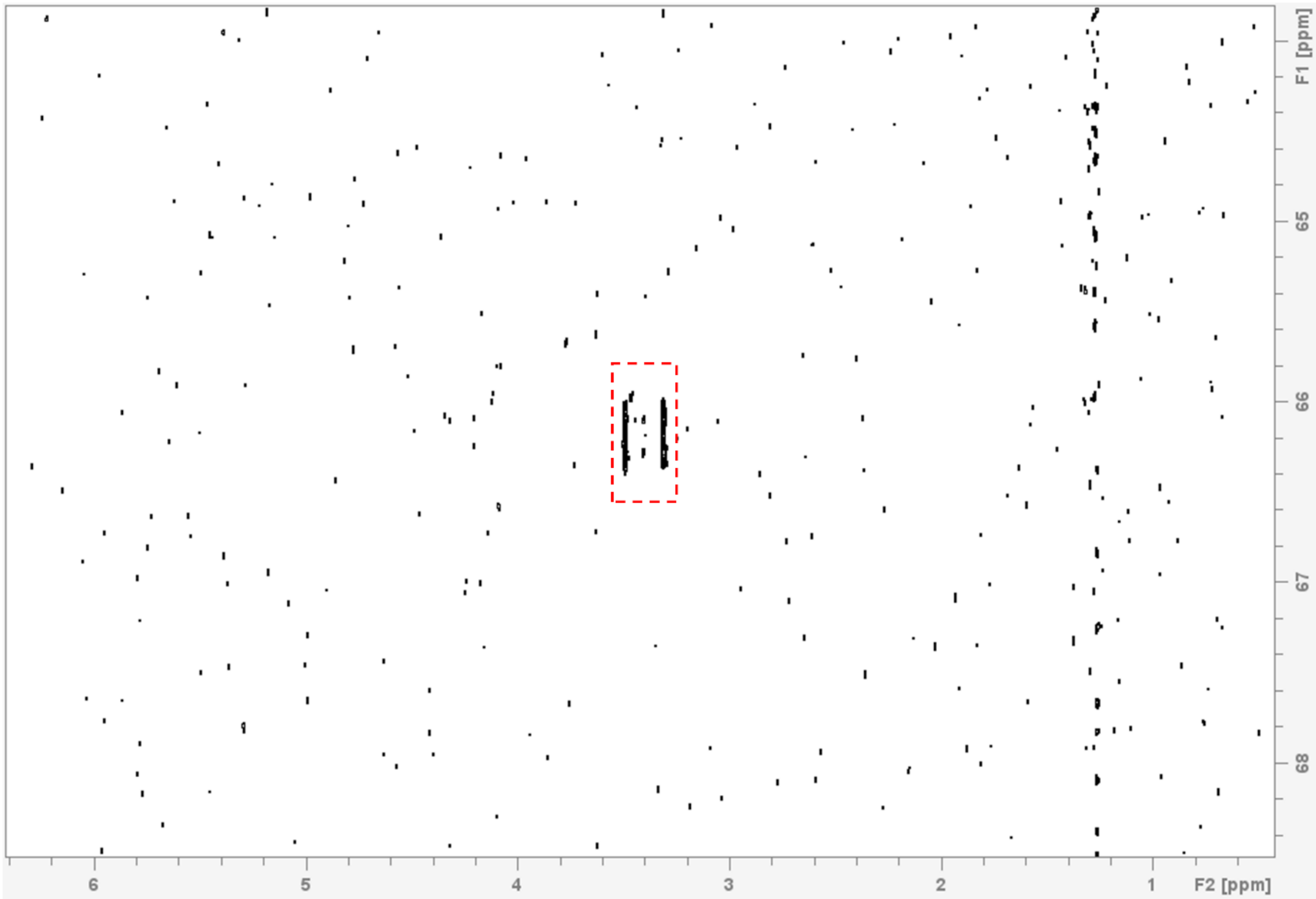

Figure S16: Full 2D HMBC spectrum corresponding to Figure 3A (Hep3B). The dashed box indicates the region of interest.

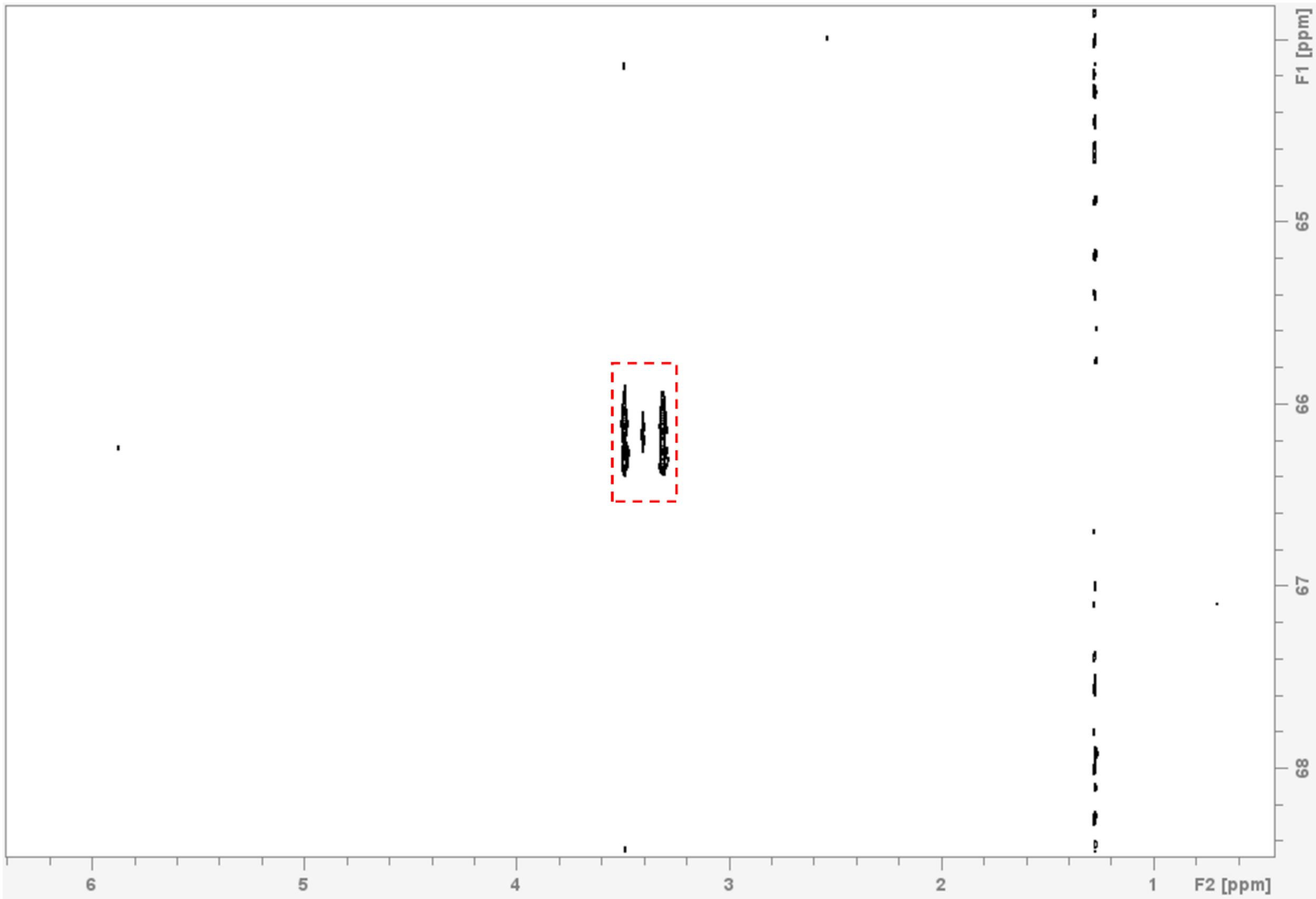

Figure S17: Full 2D HMBC spectrum corresponding to Figure 3A (NKNT-3). The dashed box indicates the region of interest.

Table S3: HMBC peak assignment

**PtdCho substrate**	**HMBC (H→C)**	**δ_C (ppm), mult**	**δ_H (ppm), mult (J)**
Etha	$N^+(C\mathbf{H}_3)_3 \rightarrow \mathbf{C}H_2N^+$	66.2, d ($^1J_{CC} \approx 40$ Hz)	3.40, s
Cho	$N^+(C\mathbf{H}_3)_3 \rightarrow \mathbf{C}H_2N^+$	66.2, d ($^1J_{CC} \approx 40$ Hz)	3.40, d ($^3J_{CH} \approx 140$ Hz)

References

1. Lapin, J. and A.A. Nevzorov, *De novo NMR pulse sequence design using Monte-Carlo optimization techniques.* J Magn Reson, 2020. **310**: p. 106641.
